# Natural history study of glycan accumulation in large animal models of GM2 gangliosidoses

**DOI:** 10.1371/journal.pone.0243006

**Published:** 2020-12-01

**Authors:** Catlyn Cavender, Linley Mangini, Jeremy L. Van Vleet, Carley Corado, Emma McCullagh, Heather L. Gray-Edwards, Douglas R. Martin, Brett E. Crawford, Roger Lawrence

**Affiliations:** 1 Research, BioMarin Pharmaceutical Inc., Novato, CA, United States of America; 2 University of Massachusetts Medical School, Worcester, MA, United States of America; 3 Scott-Ritchey Research Center and Department of Anatomy, Physiology, and Pharmacology, Auburn University College of Veterinary Medicine, Auburn, AL, United States of America; Nathan S Kline Institute, UNITED STATES

## Abstract

β-hexosaminidase is an enzyme responsible for the degradation of gangliosides, glycans, and other glycoconjugates containing β-linked hexosamines that enter the lysosome. GM2 gangliosidoses, such as Tay-Sachs and Sandhoff, are lysosomal storage disorders characterized by β-hexosaminidase deficiency and subsequent lysosomal accumulation of its substrate metabolites. These two diseases result in neurodegeneration and early mortality in children. A significant difference between these two disorders is the accumulation in Sandhoff disease of soluble oligosaccharide metabolites that derive from N- and O-linked glycans. In this paper we describe our results from a longitudinal biochemical study of a feline model of Sandhoff disease and an ovine model of Tay-Sachs disease to investigate the accumulation of GM2/GA2 gangliosides, a secondary biomarker for phospholipidosis, bis-(monoacylglycero)-phosphate, and soluble glycan metabolites in both tissue and fluid samples from both animal models. While both Sandhoff cats and Tay-Sachs sheep accumulated significant amounts of GM2 and GA2 gangliosides compared to age-matched unaffected controls, the Sandhoff cats having the more severe disease, accumulated larger amounts of gangliosides compared to Tay-Sachs sheep in their occipital lobes. For monitoring glycan metabolites, we developed a quantitative LC/MS assay for one of these free glycans in order to perform longitudinal analysis. The Sandhoff cats showed significant disease-related increases in this glycan in brain and in other matrices including urine which may provide a useful clinical tool for measuring disease severity and therapeutic efficacy. Finally, we observed age-dependent increasing accumulation for a number of analytes, especially in Sandhoff cats where glycosphingolipid, phospholipid, and glycan levels showed incremental increases at later time points without signs of peaking. This large animal natural history study for Sandhoff and Tay-Sachs is the first of its kind, providing insight into disease progression at the biochemical level. This report may help in the development and testing of new therapies to treat these disorders.

## Introduction

β-N*-*acetylhexosaminidase (Hex) is vital in hydrolyzing and degrading glycoconjugates such as glycosphingolipids and glycoproteins that enter the lysosome [1–3]. The enzyme acts by cleaving the terminal *N-*acetylhexosamine (HexNAc), either N-acetylglucosamine (GlcNAc) or N-acetylgalactosamine (GalNAc) at the non-reducing end of glycoconjugates and their metabolites. Hex is encoded by two genes, *HEXA* and *HEXB*. *HEXA* encodes the α-subunit of the enzyme, which is directly responsible for hydrolyzing negatively charged substrates due to its positively charged binding pocket. In contrast, *HEXB* encodes the β-subunit providing substrates with a negative binding pocket [1, 4, 5]. The enzyme is only functional upon dimerization and has three possible isoforms: heterodimer Hex A (αβ**)**, homodimer Hex B (ββ), and homodimer Hex S (αα) [1, 5]. In humans, Hex A is the sole isozyme responsible for hydrolysis of GM2 gangliosides [6]. It works in conjunction with a small accessory protein, GM2 activator protein (GM2AP). GM2AP acts as a cofactor by binding to the carbohydrate and lipid portions of the GM2 ganglioside and presenting the terminal HexNAc to Hex A for hydrolysis [1, 7, 8]. The Hex B homodimer is important as well, degrading other terminal HexNAc-containing substrates that enter the lysosome. Hex S is thought to have little physiologic relevance; it is unstable and therefore plays little or no role in degrading substrates [1, 2].

Mutations in either of the *HEX* genes or the *GM2A* gene can increase substrate accumulation in patients leading to serious life-threatening disease resulting from the lysosomal accumulation of Hex substrates. These diseases are called GM2 gangliosidoses because of the large accumulation of GM2 ganglioside in patients which leads to neurotoxicity and demyelination [1]. Disease onset and severity are influenced by the amount of residual β-hexosaminidase enzyme activity, which may depend on which gene is mutated and the type of mutation [1, 9, 10]. Depending on which gene is mutated, a patient can be categorized into one of the three GM2 gangliosidoses.

Tay-Sachs disease is caused by mutations in the *HEXA* gene, leading to a deficiency in the α-subunit and consequently the biosynthesis and activity of the Hex A dimer. Hex B is not affected in Tay-Sachs; therefore, glycosphingolipids accumulate while oligosaccharide substrates continue to be cleared by the action of Hex B [1–3, 10]. Tay-Sachs disease can be further divided into infantile, juvenile, and late-onset forms depending on the age of symptom onset (3 to 5 months, 1.5 to 10 years, and older than 10 years, respectively). Patients with the infantile form experience premature death before the age of 5, and juvenile patients before the age of 15. Patients with the late-onset form experience less severe symptoms including muscle weakness, ataxia, seizures, and slurred speech, but otherwise may have a normal lifespan [2, 11].

Sandhoff disease is caused by mutations in the *HEXB* gene, resulting in a deficiency in both Hex A and Hex B enzymes [3]. Because both Hex A and Hex B are affected in this disorder, no terminal HexNAc-containing substrates are degraded. Sandhoff patients develop symptoms similar to that of Tay-Sachs and also experience early mortality due to the substrate accumulation [1, 2, 9, 12].

The third type of GM2 gangliosidosis is the AB variant, which is extremely rare and is caused by a deficiency in the GM2 activator protein, GM2AP [3, 13]. Although Hex A and Hex B enzymes are functional and remain at normal levels, lack of the activator protein results in GM2 gangliosides not being presented properly to the Hex A enzyme resulting in the pathological accumulation of gangliosides similar to that observed in Tay-Sachs.

Mutations in any one of these three genes, *HEXA*, *HEXB* or *GM2A*, can cause the accumulation of the GM2 gangliosides and other terminal HexNAc-containing substrates, which make them relevant biomarkers of disease progression and treatment effects in GM2 gangliosidosis. Other biomarkers that have been evaluated in GM2 gangliosidoses include glycoprotein-derived soluble oligosaccharides, and phospholipids [12, 14, 15].

GM2 gangliosides are glycosphingolipids (GSLs) found abundantly in neurons and throughout the CNS, but accumulate in GM2 gangliosidosis patients [4, 7]. GSLs are important in creating stable synaptic connections for memory formation and overall development of the brain [4, 7]. GM2 ganglioside accumulation results in significant CNS neuropathology including inflammation, demyelination, and neurodegeneration. One well documented symptom in patients is the cherry-red spot located in the retina [16]. Ganglioside accumulation can also lead to secondary insult to the periphery system [1].

Another substrate biomarker class includes soluble oligosaccharide metabolites which are the products of glycoprotein degradation. The Hex B homodimer plays an important role in the degradation of both N*-* and O*-*linked glycans by cleaving HexNAc at the non-reducing end of these structures [1]. Free glycans have been found to accumulate in Sandhoff patients, but not to significant levels in Tay-Sachs patients [1, 17].

A secondary storage material that accumulates alongside terminal HexNAc-containing substrates are lysosomal phospholipids called bis-(monoacylglycero)-phosphate, or BMP phospholipids. BMP phospholipids are responsible for the degradation, recycling, and chaperoning of molecules into and out of the lysosome [10, 18]. BMP phospholipids have been shown to increase upon lysosomal stress due to accumulation of incompletely degraded metabolic products, with the most abundant species being di-22:6-bis(monoacylglycerol)phosphate (BMP(22:6)). BMP(22:6) is a known biomarker for phospholipidosis and is used in assessing presence or progression of some lysosomal storage diseases [18] including Sandhoff disease [19].

Though the precise pathogenic mechanisms in Sandhoff and Tay-Sachs disease are not fully understood, disease-related storage material is known to cause grossly distended neurons with structural abnormalities such as meganeurites (swellings of the axon hillock) and ectopic neurites (small extensions from neuronal processes) [20]. Such defects may contribute to abnormal neurotransmission or other dysfunction. The accumulation of substrate metabolites is thought to cause a generalized lysosomal dysfunction leading to the accumulation of secondary substrates (e.g., cholesterol) unrelated to the primary enzyme deficiency [21]. Also, intracellular signaling cascades and the unfolded protein response may be disrupted by excessive gangliosides, which are thought to participate in signal transduction [22]. In the aggregate, the accumulation of these storage products correlates with cellular pathophysiology and likely plays an important role in neuronal insult resulting in the profound neurodegeneration observed in these disorders [23, 24].

A comprehensive natural history study of these metabolic substrates and disease-related biomarkers has not been done in large animal models for either Sandhoff or Tay-Sachs disease. These larger animal models are arguably more translatable to humans than are smaller organisms such as rodent models. For example, unlike humans, mice have an alternative catabolic pathway capable of degrading GM2 ganglioside through the action of Hex B instead of Hex A [25]. This helps reduce the ganglioside accumulation and may contribute to the less severe phenotype seen in Tay-Sachs mice compared to affected humans [3, 26]. Large animal models such as cats and sheep have a brain size and organization more similar to humans than do mice. Consequently, understanding how these biomarkers behave in large animal models could lead to a better understanding of patient disease progression at the biochemical level. Thus, we carried out a natural history study in two large animal models of GM2 gangliosidoses, Sandhoff cats and Tay-Sachs sheep. Using liquid chromatography/tandem mass spectrometry (LC-MS/MS), we measured levels of gangliosides, BMP(22:6) phospholipid, and free glycans in tissues and biological fluids from affected animals at different ages over their life spans.

## Materials and methods

The A2G0 N-glycan was purchased from Prozyme (Hayward, CA). Endoglycosidase Endo S was purchased from New England Biolabs (Ipswich, MA, USA). Natural abundance aniline and [^13^C_6_] aniline, sodium cyanoborohydride (NaBH_3_CN), were purchased from Sigma Aldrich (Milwaukee, WI, USA). Hypersep Hypercarb PGC SPE cartridges (25 mg, 1 mL) and Pierce BCA Protein Assay Kit were purchased from ThermoFisher Scientific (Waltham, MA, USA). Creatinine Assay Kit was purchased from Sigma (St. Louis, MO, USA). The ganglioside standards (GM1, GA1, GM2, GA2 and GM3) were all purchased from Enzo Life Sciences (Farmingdale, NY, USA) while the BMP(14:0) phospholipid was purchased from Avanti Polar Lipids (Alabaster, AL, USA). The 10kDa centrifugal filters were from VWR (Radnor, PA, USA). All other chemicals were of reagent or LC-MS grade for chromatography systems.

### Animals and sample collection

Sandhoff cats, Tay-Sachs sheep and species-specific age matched controls were purpose-bred from the GM2 gangliosidosis research colonies at Auburn University. Cat studies consisted of 9 wild type, 9 affected animals and sheep studies consisted of 8 wild type, 8 affected animals as indicated in S1 Table. All animal procedures were approved by the Auburn University Institutional Animal Care and Use Committee. All animals were housed, fed, watered and enriched according to IACUC-approved protocols for GM2 gangliosidosis research colonies at Auburn University. Animals were monitored at least once daily by interactions with animal care staff, graduate students and veterinarians on the project. Any welfare concerns were addressed by the veterinarian investigators on the protocol, the project veterinarian and/or the unit attending veterinarian. The unit attending veterinarian inspected animals at least once weekly. These animals were not treated with any experimental therapeutics and simply were monitored through their disease course until the pre-determined time point for euthanasia.

Cats were group housed in indoor, temperature- and light-controlled wards; were given dry and wet food from respected vendors; and were enriched by a variety of methods including trained animal care workers who socialize animals beginning when they are kittens. Cats were humanely euthanized by first being tranquilized with ketamine (10–20 mg/kg) followed by intravenous (IV) pentobarbital overdose (100 mg/kg).

Sheep were maintained as a flock in fenced grass pastures that contained shelters from sun and rain. Their grass diet was supplemented with grain, minerals and hay as needed, and freshwater containers were provided. Enrichment included normal flock behavior of sheep (including open pastures to graze and run), shearing of wool at least once per summer and daily interactions with animal care staff and/or project investigators. Sheep were humanely euthanized by first being tranquilized with a combination of midazolam (4mg/kg) and ketamine (10-20mg/kg) followed by IV pentobarbital overdose (100mg/kg).

Tissue and liquid samples were collected and frozen according to standard methods. Sandhoff cat samples were obtained from one, two and four-month-old animals, and Tay-Sachs sheep samples were obtained from three, six and nine-month-old animals. The final time point coincides with the humane endpoint of each model.

### Sample homogenization

Individual brain samples were homogenized in 1 mL of Milli-Q (MQ) water with 2.8 mm ceramic beads using a Bead Ruptor 24 Homogenizer (Omni International, Kennesaw, GA, USA) for one cycle of 20 seconds at 4.85 m/s. Protein concentrations for brain, cerebral spinal fluid (CSF), serum and plasma were determined using a BCA protein assay kit per manufacturer’s instructions. Protein concentrations were used to standardize each sample. Creatinine concentrations for urine were determined using a creatinine assay kit per manufacturer’s instructions. Creatinine concentrations were used to normalize urine samples. Homogenized samples were split for ganglioside and glycan analysis.

### Ganglioside and BMP preparation and analysis

Homogenized tissue samples were diluted to 4 μg protein/μL homogenate with MQ water based on the BCA protein assay results. An amount of sample equal to 200 μg of protein was extracted with 500 μL 95/5 methanol/glacial acetic acid (v/v). Extracts were filtered through 10 kDa centrifugal filter and clarified samples were injected into the LC-MS/MS or stored at 20°C until further use. The five different gangliosides and BMP(22:6) were analyzed in each sample with an Acquity UPLC and Xevo TQ-S Micro Triple Quadrupole Mass Spectrometer (Waters Corporation, Milford, MA, USA).

The gangliosides were separated using an Acquity UPLC Glycan BEH Amide column (Waters Corporation, Milford, MA, USA). Mobile phase A consisted of 5 mM ammonium acetate in 94.5% acetonitrile, 2.5% methanol, 2.5% MQ water, with 0.5% formic acid, and mobile phase B consisted of 100% MQ water. The column temperature was set at 50°C and a flow rate of 0.4 mL/min. Sample injection volume was 2 μL. Samples were ionized in positive ion mode by ESI with the capillary voltage set at 1.0 kV, the desolvation temperature set at 500°C, and a desolvation gas flow of 1000 L/hr. The samples were eluted using a concentration gradient of 95% A/5% B for two minutes, increase to 50% A/50% B over 10 minutes, holding there for another 10 minutes, and finish at 95% A/5% B for the last four minutes. Samples were quantified based on a five-point standard curve (100–6.25 pg/μL) of GM1, GA1, GM2, GA2, and GM3 gangliosides. The sum of two parent-product ion transitions for each ganglioside (**Table 1**) was used for quantitation [27].

**Table 1:** Retention times for each analyte of interest.

Analyte	Species	Precursor Ion	Product Ion	Cone (V)	Collision Energy (V)	Approx. Retention Time (min)
GM1	GM1(36:1)	1546.7	366.1	10	36	7.2
	GM1(38:1)	1574.7	366.1	10	36	
GA1	GA1(36:1)	1255.7	366.1	10	24	6.6
	GA1(38:1)	1283.8	366.1	10	24	
GM2	GM2(36:1)	1384.7	204.1	10	44	6.6
	GM2(38:1)	1412.7	204.1	10	44	
GA2	GA2(36:1)	1093.6	264.3	10	54	5.3
	GA2(38:1)	1121.6	292.3	10	54	
GM3	GM3(36:1)	1181.5	264.3	10	54	6.1

For BMP(22:6), samples were separated on an Acquity UPLC HSS C18 column (Waters Corporation, Milford, MA, USA). Mobile phase A consisted of 5 mM ammonium formate in 74% methanol, 25% MQ water, and 1% formic acid, and mobile phase B consisted of 5 mM ammonium formate in 99% methanol and 1% formic acid. The column temperature was set at 50°C with a 5 μL injection volume at a flow rate of 0.1 mL/min. Samples were ionized in negative ion mode by ESI with the capillary voltage set at 3.5 kV, desolvation temperature set at 600°C, and the desolvation gas flow at 1000 L/hr. The samples were eluted using a concentration gradient starting at 80% A/20% B for one minute, ramping to 100% B over five minutes, holding for 10 minutes, followed with 80% A/20% B for the last four minutes. A five point standard curve (100–6.25 pg/μL) of BMP(14:0) was prepared and BMP(22:6) concentrations are reported as equivalents of BMP 14:0. One precursor to product ion transition was monitored for each BMP species (**Table 2**).

**Table 2:** Retention times for each analyte of interest.

Analyte	Precursor Ion	Product Ion	Cone (V)	Collision Energy (V)	Approximate retention Time (min)
BMP(14:0)	665.4	227.2	10	32	7.5
BMP(22:6)	865.5	327.3	10	32	7.5

### Glycan sample preparation

Homogenized and clarified tissue samples were diluted to 240 μg of protein in 1 mL of MQ water. Next, Hypersep Hypercarbon PGC were prepared by first washing three times with 1 mL of 80% acetonitrile with 0.1% TFA, followed by three times with 1 mL of MQ water. The diluted samples were applied to the column and the flow through was discarded. Columns were washed three times with 1 mL of MQ water, then placed over a 15 mL centrifuge tube. Free glycans were eluted with three 1 mL aliquots of 30% acetonitrile with 0.1% TFA. The collected free glycans were dried down in a SpeedVac overnight.

Dried free glycan metabolites were labeled by reductive amination by first reconstituting them with 17 μL of aniline, mixing 10–15 times. Next, 17 μL of freshly prepared 1 M NaCNBH_3_ in 70% DMSO and 30% glacial acetic acid was added to each sample and again mixed 10–15 times, then incubated overnight at 37°C. The labeled samples were dried in a SpeedVac overnight.

Prior to analysis by LC/MS, the labeled samples were reconstituted with 150 μL MQ water. 30 μL of each sample was placed into a mass spectrometry vial with 5 μL of [^13^C_6_] aniline tagged internal standard (A2G0′) and dried in a SpeedVac for one hour. The dried sample and internal standard were then reconstituted in 20 μl 78% acetonitrile and 22% 100 mM ammonium formate pH 4.5 for analysis.

### Internal standard preparation

In order to generate the A2G0′ hexasaccharide standard, it was necessary to first cleave off the reducing end GlcNAc residue from the fully intact A2G0 heptasaccharide. The A2G0 glycan (2000 pmoles) was digested with Endo S (800 units) in a total volume of 20 ***μ***L of 50 mM Sodium Phosphate pH 7.5 for 18–24 hours followed by purification on a Sep-PAK C18 SPE cartridge (100 mg, 1 mL), as to the manufacturer’s instructions, and dried by centrifugal evaporation. The dried resulting A2G0′ (A2G0 prime) glycan standard was then labeled with ^13^C_6_-aniline (15 ***μ***L, 15 ***μ***L 1 M NaCNBH_3_ in 70:30 DMSO:HOAc) at 37°C for 18–24 h and the remaining ^13^C_6_-aniline was removed by centrifugal evaporation. Prior to analysis, the internal standard was dissolved in water.

### LC/MS analysis and quantitation of A2G0′

LC/MS analysis was performed on an Acquity UPLC system equipped with a Glycan BEH Amide HILIC column (1.7 ***μ***m, 2.1 mm x 150 mm) (Waters, Milford, MA, USA) connected to a Thermo LTQ Orbitrap XL mass spectrometer. Solvent A was 100 mM Ammonium Formate pH 4.5 and Solvent B was Acetonitrile with an initial composition of 22% A/78% B and a flow rate of 0.2 mL/min. The column temperature was kept at 60°C. Prior to injection an amount of aniline labeled sample equal to 70 ***μ***g of protein, for tissues, or 20 ***μ***L, for urine, was placed in a LC/MS sample vial along with 10 pmoles of ^13^C_6_-aniline labeled A2G0′ biomarker internal standard and dried by centrifugal evaporation. The samples were then dissolved in a solution of 22% A/78% B. The labeled free glycans were eluted using a gradient profile of 22% A/78% B to 37% A/63% B over 65 min, 100% A/0% B for 6 min, 100% A/0% B to 22% A/78% B in 5 min and held there for 9 min.

The LTQ Orbitrap XL was operated in the positive ion mode and the sample introduced by ESI. The capillary temperature was set at 250°C with a spray voltage of 2 kV. The sheath gas flow was set to 58 and the auxiliary gas flow to 9. Full scans were performed at a resolution of 60,000 and a range of 200–2250 m/z. Data-dependent collision induced dissociation (CID) was performed on the three most intense ions in the full scan using a normalized collision energy of 35 V and an activation time of 30 ms. The N-glycan A2G0′ metabolite was determined by ratio-metric comparison of the ^12^C_6_-aniline labeled endogenous A2G0′ ion abundance with that of the known molar amount of the internal standard spike as previously described [28].

### Statistical analysis

Statistical analyses were performed using R software (version 3.6.3, R Foundation for Statistical Computing, Vienna, Austria [29]). All data points were included in the analyses with no outliers excluded. For comparing analyte differences between affected and unaffected animals at each time point, one-tailed, unpaired Student’s *t* test was used. For comparing analyte levels at different time points within groups, two-way ANOVA followed by Tukey correction was performed. For correlations between ganglioside and A2G0′ levels, a Pearson correlation test was used. All data are expressed as mean ± SD throughout the text and figures.

## Results

### CNS accumulation of gangliosides in a cat model of Sandhoff disease

For our hexosaminidase β-subunit deficiency longitudinal study, we used a feline model of Sandhoff disease with a spontaneous mutation in the *HEXB* gene [30]. Residual hexosaminidase activity in this model was previously measured at approximately 1% of normal on average across the entire brain and less than 1% of normal in the occipital lobe [31]. Age-matched unaffected normal control cats were used for comparison to Sandhoff cats at ages of one, two and four months (the humane endpoint) for substrate accumulation analysis. Single brain punch samples taken from the occipital lobe of each cat were homogenized, lipid extracted with acidified methanol and gangliosides quantified using LC-MS/MS analysis as described in the Method section. We measured the levels of two GM2 ganglioside isomer species, GM2 (36:1) and GM2 (38:1) which are known to accumulate in *HEXB* null mice [19, 27, 32] and human patients [12, 33]. We also measured levels of GM1 and GM3 gangliosides as well as the corresponding asialo species for GM1 and GM2 (GA1 and GA2).

**Fig 1:**
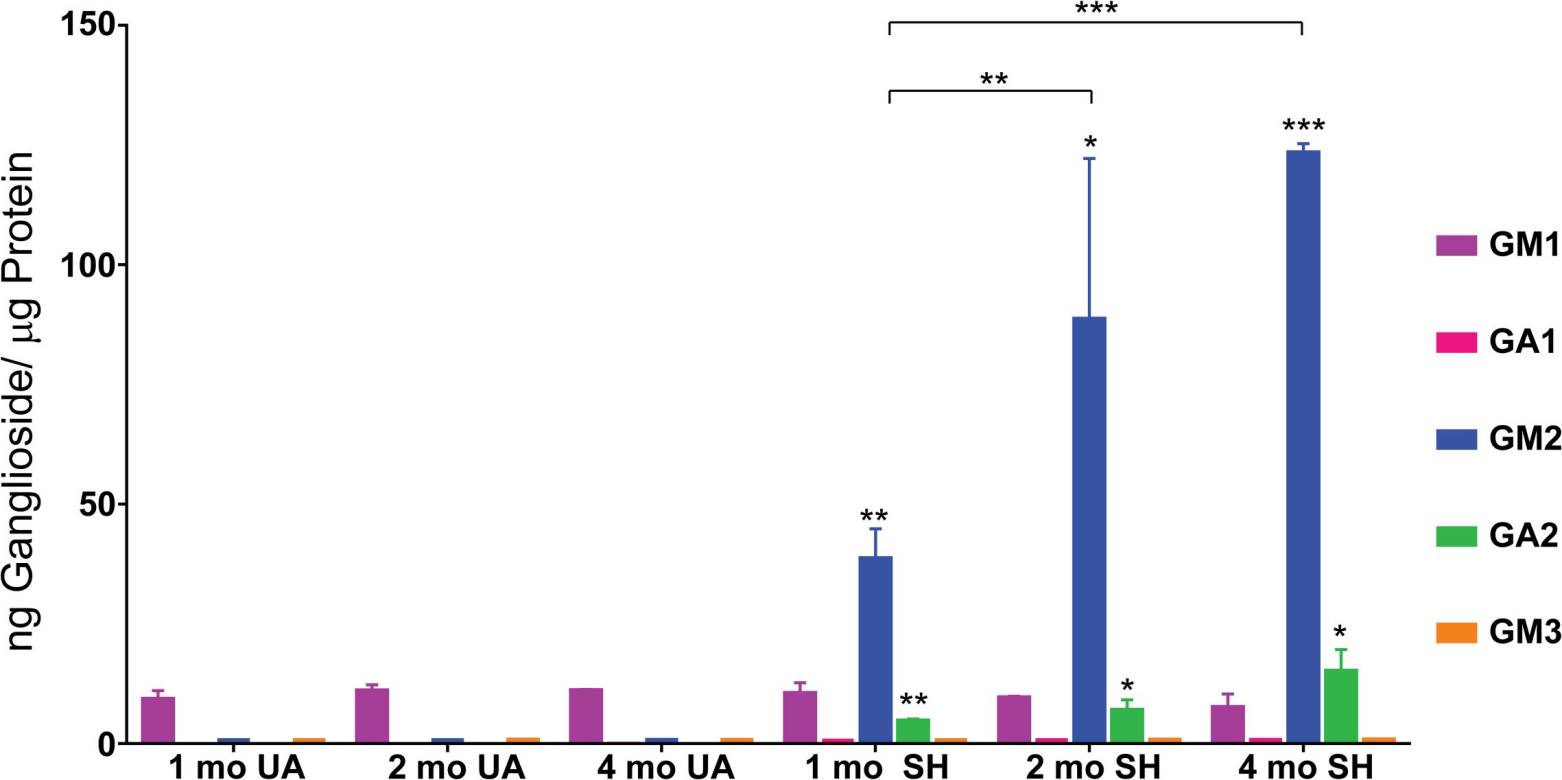
Feline Sandhoff brain ganglioside levels. Ganglioside levels were measured in Sandhoff (SH) and age-matched unaffected (UA) cats. Samples from biopsied occipital lobe taken from animals 1, 2, and 4 months old (mo) were analyzed and the mean results for each time point (n = 3) are shown as ng Ganglioside/μg protein equivalents. Error bars represent ± SD. The degree of significant difference between age-matched UA and SH felines is indicated by the number of asterisks above each bar for GM2 and GA2 (*** p ≤ 0.001, ** p ≤ 0.01, * p ≤ 0.05). Square brackets with asterisks above GM2 bars indicate the significant difference in GM2 levels between time points for affected cats.

Our results for the occipital lobe showed GM1 gangliosides were at similar baseline levels (between 7.1 and 10.6 ng GM1/μg protein) across all the wild type and Sandhoff cat CNS samples (**Fig 1 and S2 Table**). Little to no GA1 or GM3 gangliosides were detected in either affected or unaffected samples. In sharp contrast, GM2 ganglioside levels in the one-month-old Sandhoff cats were greatly elevated nearly 250-fold compared to age-matched normal controls (0.17 vs 38.2 ng GM2/μg protein, **Fig 1 and S2 Table**). The corresponding asialo GA2 gangliosides were detected in the one month old controls at very low levels (0.02 ng GA2/μg protein) but were detected at elevated levels (4.3 ng GA2/μg protein) in the age-matched affected felines (**Fig 1 and S2 Table**). Like the increase seen with GM2, this also constitutes a nearly 250-fold increase in detectable levels related to disease. Furthermore, unlike what we observed for the age matched normal controls, the levels of both GM2 and GA2 gangliosides were detected at higher levels at later time points consistent with levels continuing to rise in affected animals as they advance in age (**Fig 1 and S2 Table**). GM2 levels increased from 38.2 ng GM2/μg protein to 88.2 ng GM2/μg protein by two months of age and then increased again to 122.9 ng GM2/μg protein by the last time point at four months. GA2 levels increased from 4.3 ng GA2/μg protein to 6.6 ng GA2/μg protein by two months of age and then again to 14.7 ng GA2/μg protein by four months. ANOVA analysis showed these progressive increases to be significant (**Fig 1 and S3 Table**) and the measured trends showed no signs of slowing down. Since we did not observe a drop in the rate of substrate accumulation by the last time point tested, these results may have implications for the severity of disease as affected animals continue to age and accumulate a greater ganglioside burden.

### CNS accumulation of BMP phospholipids in a feline model of Sandhoff disease

**Fig 2:**
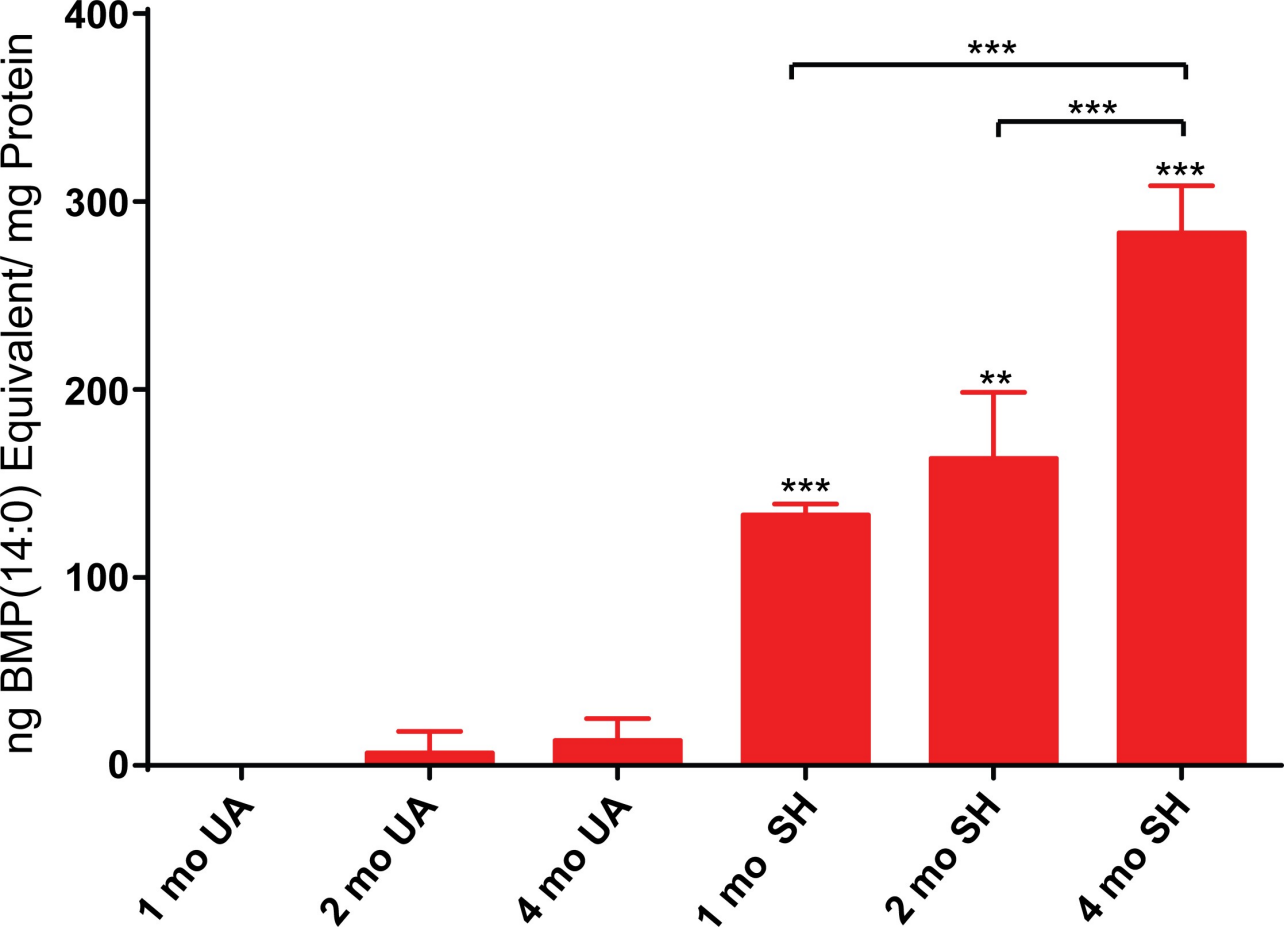
Feline Sandhoff brain BMP(22:6) levels. BMP phospholipid levels were measured in Sandhoff (SH) and age-matched unaffected (UA) cats. Samples from biopsied occipital lobe taken from animals 1, 2, and 4 months old (mo) were analyzed and the mean results for each time point (n = 3) are shown as ng BMP(14:0) phospholipid equivalent/mg protein equivalents. Error bars represent ± SD. The degree of significant difference between age-matched UA and SH felines is indicated by the number of asterisks above each bar (*** p ≤ 0.001, ** p ≤ 0.01, * p ≤ 0.05). Square brackets with asterisks above bars indicate significant difference in BMP(22:6) levels between time points for affected cats.

After measuring ganglioside levels in occipital lobe samples taken from normal control and affected cats, we analyzed these samples for the secondary phospholipid biomarker BMP (22:6) previously shown to be elevated in Sandhoff disease [19]. As expected, BMP(22:6) was found at elevated levels in affected animals compared to age matched normal controls which exhibited little to no BMP(22:6) (**Fig 2 and S2 Table**). At the one month time point, no BMP(22:6) was detected in the age-matched control while 133.3 ng BMP(22:6)/mg protein was detected in the affected cats. These phospholipid levels increased slightly to 163.3 ng BMP(22:6)/mg protein by the two month time point but this increase over that detected at one month was not significant (**Fig 2, S2 and S3 Tables**). Levels increased further to 283.3 ng/mg protein by the four-month time point. The increase in BMP(22:6) between the two earlier time points and the last was significant (**S3 Table**) demonstrating that, like GM2 and GA2 gangliosides, BMP(22:6) phospholipid levels in the brain follow an increasing trend with age. We also saw no signs of this trend slowing down by the last time point tested similar to the age dependent accumulation seen for GM2 and GA2 gangliosides in these affected animals (**Fig 1)**. Thus, Sandhoff cats are subjected to an increasing burden of both gangliosides and BMP phospholipids over time which may have a significant impact on disease severity as these animals get older.

### Accumulation of hexosaminidase substrate oligosaccharide metabolites in a feline model of Sandhoff disease

The β-subunit of hexosaminidase is important for the degradation of both gangliosides and other glycoconjugates such as proteoglycans and glycoproteins [34, 35]. Thus, Hex B deficiency leads to the accumulation of multiple substrate metabolites including soluble oligosaccharides as has been observed in a number of previous studies [14, 36–39].

**Fig 3:**
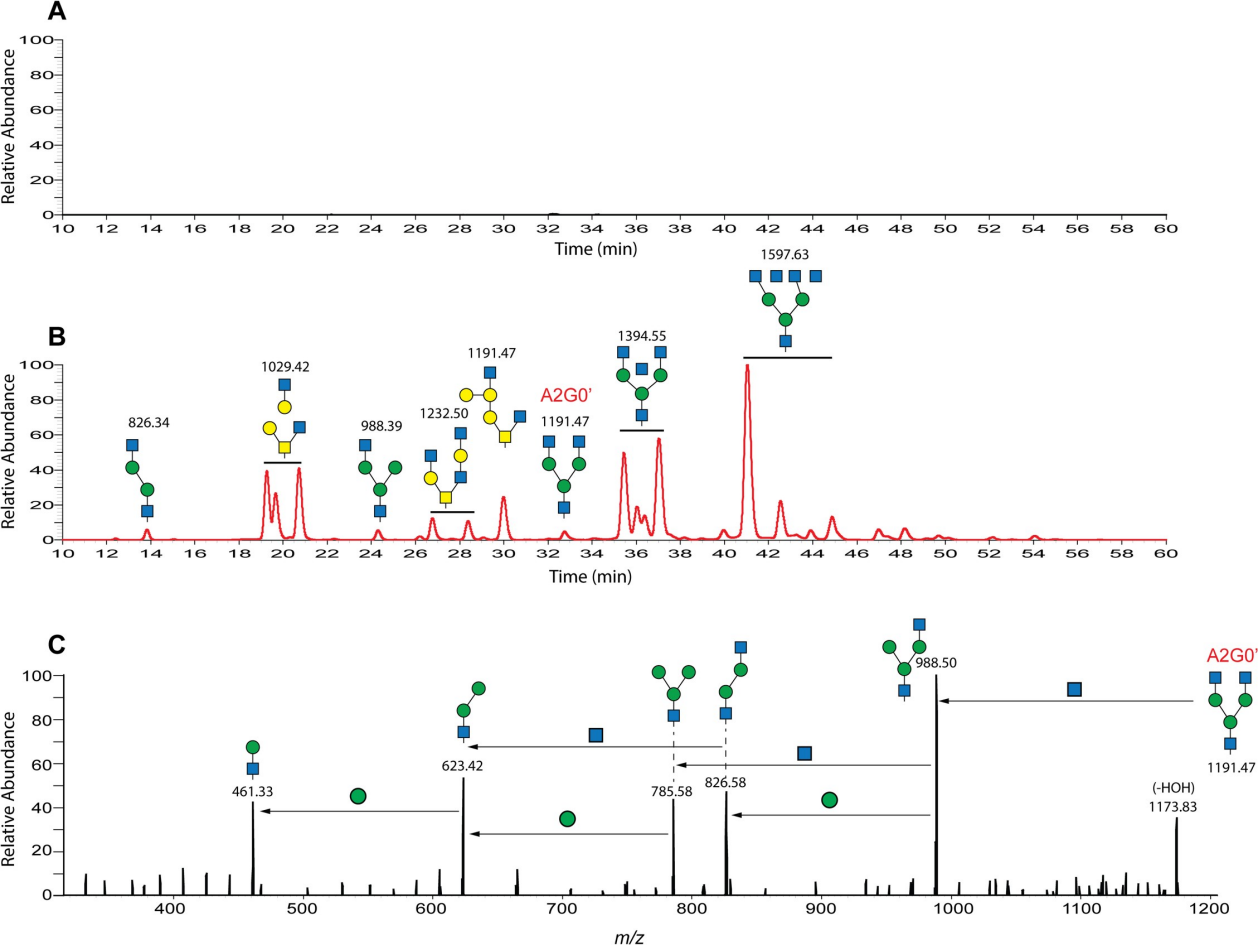
Soluble oligosaccharide metabolites in Sandhoff and unaffected cat brains. The soluble fraction from water homogenates made from occipital lobe samples collected from both wild type and Sandhoff cats at one month of age was end-labeled with aniline as described in the Methods section in order to detect the presence of reducing sugar metabolites. Labeled products were analyzed by LC/MS with molecular species determined to be oligosaccharides by data-dependent product ion analysis. The results for both wild type and affected cats are presented at the same ion intensity scale (Relative Abundance) with the column retention time indicated on the x axis. (A) Extracted ion current for free glycans in one-month old normal unaffected control feline occipital lobe showing little or no detection of soluble oligosaccharides. (B) Extracted ion current for free glycans in a one-month old Sandhoff cat occipital lobe showing a number of soluble glycans with their corresponding *m/z* values above each peak or set of isobaric peaks (indicated by a solid line above). Putative structures based on *m/z* values and product ion analysis by CID to detect species with terminal HexNAc residues are also shown. The quantifiable hexasaccharide species with *m/z* = 1191.47, A2G0′ is indicated above the peak eluting at approximately 33 minutes. (C) Data-dependent product ion profile for the putative A2G0′ ion eluting at approximately 33 minutes. The major product ions are shown along with their *m/z* values which are consistent with the structures shown. Arrows between the product ions illustrate the inter-ring cleavage loss of neutral monosaccharide fragments leading to the generation of subsequently smaller product ions. For example, the product ion with *m/z* value of 988.50 was formed by the neutral loss of a HexNAc residue by the parent ion from its nonreducing end. Monosaccharides are shown symbolically as follows: glucose (blue circles), galactose (yellow circles), mannose (green circles), N-acetylglucosamine (blue squares), and N-acetylgalactosamine (yellow squares).

In order to detect these types of metabolites in the feline CNS, we used a method we previously employed to detect and characterize similar limit digestion products in another related disease, GM1 Gangliosidosis [27]. Soluble free glycans present in water homogenates made from occipital lobe samples were end-labeled with aniline by reductive amination, separated by forward phase chromatography on an amide column and labeled glycans were detected by mass spectrometry as described in the Methods section. Based on the *m/z* values of intact molecular ions and data dependent product ion analysis, a number of glycan metabolites were detected in one-month old Sandhoff cat brains while little to no glycans were detected in age-matched unaffected normal controls (**Fig 3**). We saw similar results at all time points tested with large amounts observed in all affected animals and no significant signal detected in age-matched normal controls. Based on the Hex B deficiency in affected felines, these glycan metabolites should have in common HexNAc at their non-reducing ends consistent with their being limit digestion products. This was verified on the mass spectrometer for many of these species by product ion analysis after data dependent collision induced dissociation (CID) (see example in **Fig 3**). Product ions consistent with the neutral loss of HexNAc from the non-reducing end allowed us to assign putative structures to many of these species (**Fig 3B**). One of these glycan metabolites is a hexasaccharide with a *m/z* value (1191.47) consistent with an aniline labeled metabolite derived from the bi-antennary complex N-glycan GlcNAc-Man- (GlcNAc-Man)-Man-GlcNAc-GlcNAc (A2G0, Oxford notation). This metabolite (see **Fig 3B**) differs from the parent glycan by the loss of the reducing-end GlcNAc residue which occurs before the β-hexosaminidase catalyzed step in the catabolic pathway for N-linked glycan degradation and is therefore not affected by Hex A/B deficiency [40]. For clarity, this metabolite is referred to in this paper as A2G0′ (A2G0 prime). A standard for this metabolite, useful for its quantitation by glycan reductive isotope labeling LC-MS (GRIL-LC/MS) [28], can be prepared from commercially available A2G0 by enzymatic cleavage of the reducing end GlcNAc and subsequent differential isotope labeling with aniline as described in the Methods section. For quantitation, we spiked a known molar amount of [^13^C_6_] aniline-labeled A2G0′ internal standard into each sample after labeling the free glycans in them with [^12^C_6_] aniline. The quantity of A2G0′ in the sample was determined by comparing the ion intensity of endogenous A2G0′ with that of the internal standard. Brain homogenates, CSF, plasma, and urine samples from both affected and age-matched unaffected control animals were analyzed for this free glycan as described in the Methods section.

**Fig 4:**
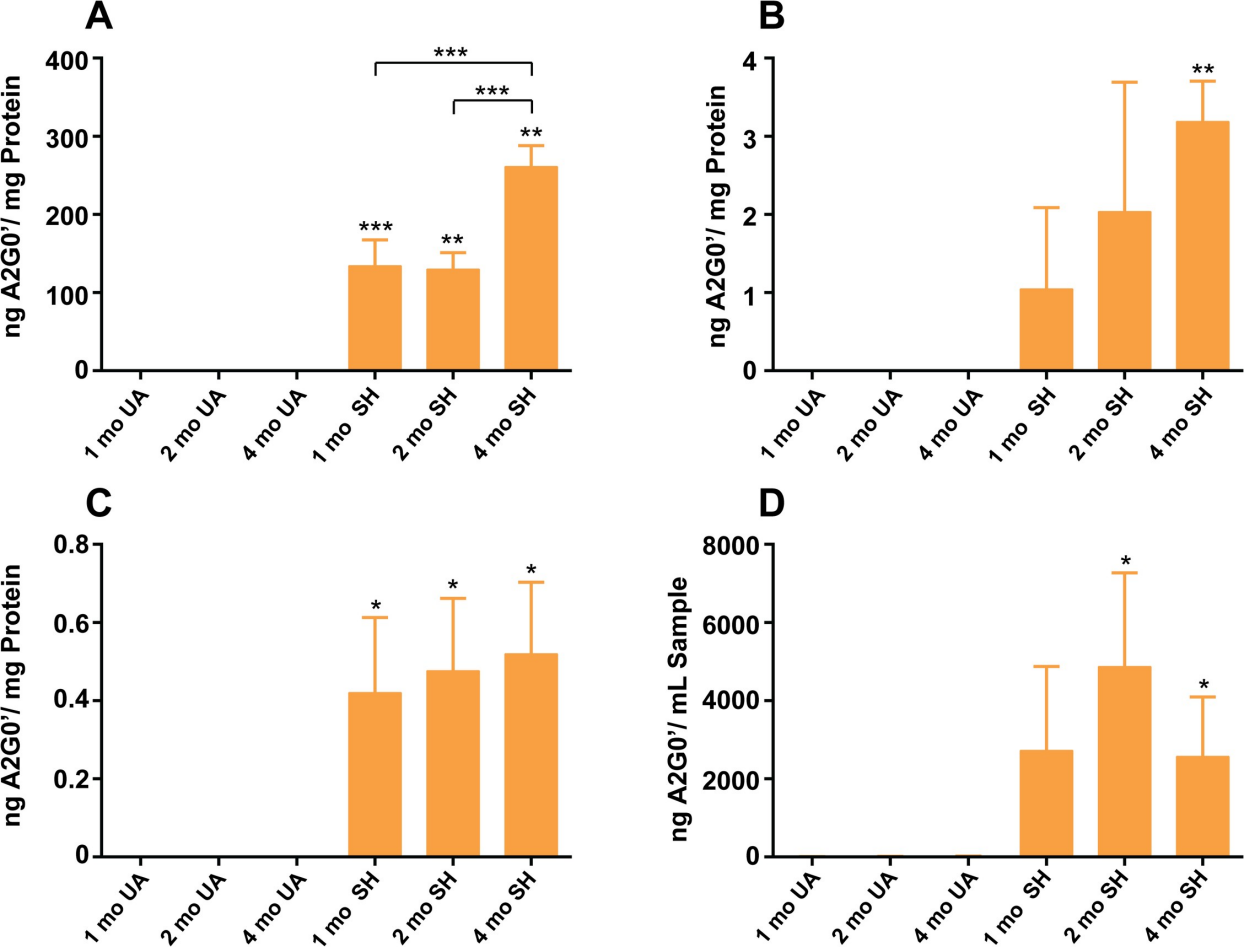
Feline Sandhoff A2G0′ glycan metabolite levels. Samples from Sandhoff (SH) and unaffected (UA) age-matched cats 1, 2, and 4 months old (mo) were analyzed with the mean results shown for each time point (n = 3) as ng A2G0′/mg protein equivalents or ng A2G0′/mL sample. Error bars represent ± SD. The degree of significant difference between age-matched UA and SH felines is indicated by the number of asterisks above each bar (*** p ≤ 0.001, ** p ≤ 0.01, * p ≤ 0.05). Square brackets with asterisks above bars indicate significant difference in A2G0′ levels between time points. (A) Mean glycan levels in occipital lobe. (B) Mean glycan levels in cerebral spinal fluid (CSF). (C) Mean glycan levels in plasma. (D) Mean glycan levels in urine (not normalized to creatinine).

Our analysis of A2G0′ in both affected and unaffected Occipital lobe samples showed no detectable signal for this analyte in normal controls at all time points tested while affected animals exhibited large amounts of A2G0′ (**Fig 4A and S2 Table**). At the one-month time point, A2G0′ levels were 134.2 ng/mg protein with levels not significantly changing by the two-month time point (129.8 ng/mg protein). However, by the last time point at four months, A2G0′ levels had increased to 261.2 ng/mg protein, a two fold increase from the levels seen at the earlier time points (**Fig 4A and S3 Table**). Thus, we observed evidence for an age-related increase for this glycan metabolite in affected brain samples consistent with what we found for the other analytes we tested. Also, because we were unable to detect A2G0′ in any of the age-matched normal controls, this metabolite is a good candidate for a Sandhoff disease-related biomarker in the brain, similar to other β-hexosaminidase substrates such as GM2 and GA2 gangliosides.

**Fig 5:**
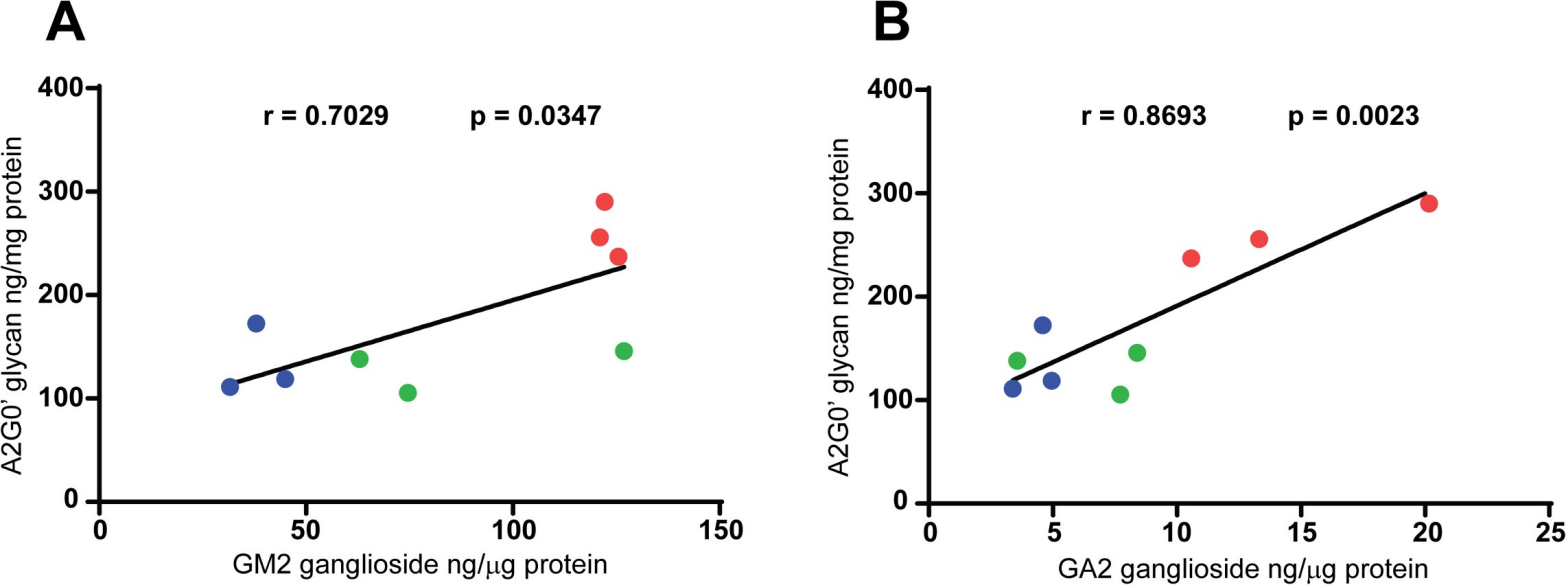
Correlation between A2G0′ and GM2/GA2 ganglioside accumulation. The data from Figs 1 and 4 for brain (occipital lobe) homogenate levels of GM2 and GA2 gangliosides and A2G0′ glycan metabolite are plotted against each other showing a good positive correlation between each ganglioside and the free glycan. Both the Pearson correlation coefficient (r) and p value (p) are shown. The data for all time points: 1 month of age (blue), 2 months of age (green) and 4 months of age (red) for affected animals and all age matched unaffected normal animals (black) are shown. (A) Correlation between GM2 ganglioside and A2G0′ glycan levels in brain homogenates. (B) Correlation between GA2 ganglioside and A2G0′ glycan levels in brain homogenates.

The pattern of disease-related accumulation for A2G0′, showing no significant changes in accumulation between the first and second time points but a significant increase seen by the last time point, is similar to that of GA2 gangliosides and BMP(22:6) phospholipid, suggesting similar rates of increase for these three analytes. The pattern of increase for GM2 gangliosides differed from this trend by showing a significant increase by the intermediate time point in affected animals. While the pattern of increase may differ among some of the analytes tested, they all showed the same trend of increasing with age in affected cats. When we compared the increasing trends for gangliosides and A2G0′, we observed good positive correlations between A2G0′ and both GM2 and GA2 ganglioside levels in the occipital lobe of the affected animals (**Fig 5**). In addition to exhibiting age-related increases, none of the analytes showed signs of reaching a plateau suggesting that these pathologically relevant molecules can continue to accumulate, possibly increasing the severity of the disease as the animals mature.

We next analyzed a series of fluid samples, collected from the same animals that the brain samples were taken from, which included cerebral spinal fluid (CSF), plasma, and urine samples. Since analyzing gangliosides and phospholipids in these liquid matrices is challenging, we focused our attention on measuring A2G0′ levels which we reasoned would be easier to detect because of its soluble nature. As was true for the occipital lobe, we were unable to detect A2G0′ in any of the CSF samples collected from normal controls at any of the time points tested. However, we detected A2G0′ in affected cats with 1.0 ng/mg protein detected at 1 month of age increasing to 3.2 ng/mg protein by 4 months of age (**Fig 4B and S2 Table**). While the trend in mean values suggests a linear increase with age, there was no significant differences in these levels (**S3 Table**). Thus, the levels of accumulation in CSF appeared to have largely plateaued by the first time point. The results for plasma were similar to those for CSF (**Fig 4C and S2 Table**). Again, none of the age-matched normal controls exhibited A2G0′ while all of the affected animals had detectable levels, albeit, at generally lower levels than those seen in CSF. Levels ranged from 0.42 ng/mg protein at 1 month to 0.52 ng/mg protein by 4 months, though this marginal increase was not significant (**S3 Table**). The results for urine differed from the results for the other matrices primarily because of the unaffected controls which exhibited measurable levels of A2G0′ (**Fig 4D and S2 Table**). At the one-month time point, A2G0′ was measured in normal control cats at 4.7 ng/mL urine. This level increased to 19.5 ng/mL urine by the terminal time point in the unaffected animals. It is important to note that the urine samples were not normalized to creatinine and therefore measurements were not adjusted for possible variations in urine concentration. In contrast, levels were much higher in affected cats. For example, urine A2G0′ levels at the earliest time point were measured at 2726.2 ng/mL urine, an over 500-fold increase compared to age-matched normal cats. As with plasma, we saw no significant increase in glycan metabolite levels at the later time points (**S3 Table**). Thus, all the liquid matrices differed from brain tissue by having plateaued by the one-month time point in affected animals. This suggests that in affected tissues, such as the occipital lobe, that A2G0′ is continuing to accumulate as animals age but the levels in biological fluids are in equilibrium between the cellular processes that route excess A2G0′ into these fluids and the eliminatory systems that remove it from the body.

Our results show that the levels of GM2 gangliosides and soluble oligosaccharide metabolites such as A2G0′ in a large animal model of Sandhoff disease correlate well with this disorder. Glycans in Sandhoff patients have been characterized in past research [38, 41], but this is the first account of quantitatively measuring free glycan metabolites in urine from cats with Sandhoff disease. While, the A2G0′ metabolite was detectable in all four matrices tested, its abundance in urine collected from affected animals suggests that it could be useful as a non-invasive clinical biomarker comparable to gangliosides which are harder to access outside of the brain. In fact, we observed good positive correlations between A2G0′ and both GM2 and GA2 ganglioside levels in the brains of the affected animals used in this study.

### CNS accumulation of gangliosides in an ovine model of Tay-Sachs disease

Tay-Sachs sheep have a naturally occurring spontaneous mutation in the *HEXA* gene [3, 42]. Residual HexA activity in this model organism was previously measured at approximately 8% of normal across the entire brain and 7% of normal in the occipital lobe [43]. Age-matched unaffected normal control sheep were used to compare to Tay-Sachs sheep at three, six and nine months of age for substrate and phospholipid accumulation analysis. The final time point corresponds to the humane endpoint for Tay-Sachs sheep.

GM2 and GA2 ganglioside analysis was performed and evaluated just as we did for the Sandhoff cat model in this study. However, unlike for the Sandhoff cats where we only analyzed occipital lobe samples, we analyzed biopsy samples from seven different brain regions in the Tay-Sachs sheep. These regions included two cerebellum samples, a single sample from the corona radiata, the thalamus and three cerebral cortical regions (occipital, temporal, and parietal). The samples were frozen and later homogenized, the gangliosides were extracted, and LC/MS/MS was used for quantification. The data for the individual regions were then combined to give the total brain result.

**Fig 6:**
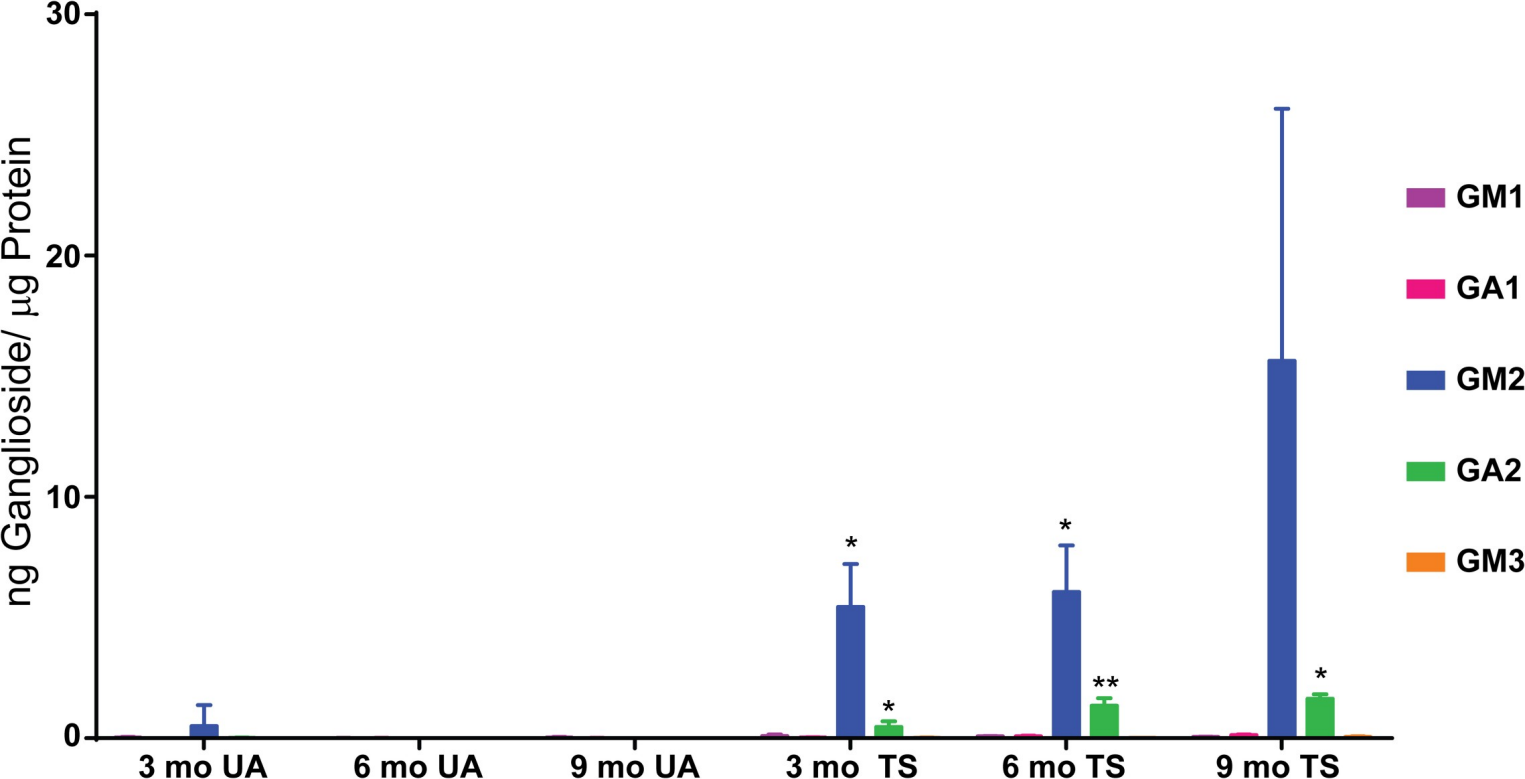
Ovine Tay-Sachs brain ganglioside levels. Tissue samples from seven different brain regions from Tay-Sachs (TS) and age-matched unaffected (UA) normal control sheep were collected at three different ages: 3, 6 (n = 3), and 9 months old (n = 2) and ganglioside levels measured by LC/MS/MS. The seven regions analyzed included two different samples from cerebellum and single biopsies from corona radiata, thalamus, occipital lobe, temporal lobe, and parietal lobe. The mean (± SD) for each set of animals for each age tested corresponding to the cumulative results from the seven different brain regions, normalized to amount protein is shown. Ganglioside levels at each time point are shown as ng ganglioside/μg protein equivalents. Error bars represent ± SD. The degree of significant difference between age-matched UA and TS sheep is indicated by the number of asterisks above each bar for GM2 and GA2 for TS sheep (*** p ≤ 0.001, ** p ≤ 0.01, * p ≤ 0.05).

Similar to our results with Sandhoff cats, our results with Tay-Sachs sheep showed little to no effects on GM1, GA1 and GM3 ganglioside levels for total brain (**Fig 6**). On the other hand, GM2 and GA2 ganglioside levels showed significant increases comparing affected animals to age-matched normal controls (**Fig 6 and S4 Table**), a trend that was observed in all regions tested (**S1 Fig**). In three-month old normal controls, we detected 0.50 ng GM2/μg protein but in age-matched affected sheep we detected greater than tenfold higher levels (5.44 ng GM2/μg protein). By the last time point, the difference between controls and affected animals was hundreds of time greater (0.009 ng GM2/μg protein and 15.6 ng GM2/μg protein, respectively) (**Fig 6 and S4 Table**). We did not observe a significant difference in GM2 levels between the three- and six-month time points in affected sheep (**S5 Table**) and unfortunately, we only had two animals in each genotypic group at the nine month time point. Thus, we were not able to conclusively determine if there was an age-related increase in GM2 levels in affected sheep as we saw with Sandhoff cats. Regardless, the higher mean value at the last time point does suggest that such an increasing trend may exist for Tay-Sachs sheep as well. Results for GA2 gangliosides exhibited significant levels of increase at all time points analyzed (**Fig 6 and S4 Table**) compared to age-matched controls and also showed significant age-related increases in affected sheep across the ages tested (**S5 Table**). Since the final time point showed the highest levels of both GM2 and GA2, brain levels appear to be continuing to increase with age, leading to a progressive substrate burden as Tay-Sachs sheep get older.

While all of the 7 regions tested from affected animals showed large relative increases in GM2/GA2 levels compared to age-matched normal controls, the trends due to age showed some distinct patterns (**S1 Fig**). Since we analyzed only the occipital lobe in the Sandhoff cat, we compared the results for occipital lobe from Sandhoff cats to those of Tay-Sachs sheep. The observed GM2 levels were approximately 18-fold less in Tay-Sachs sheep than those measured in the feline Sandhoff occipital lobe (**S2 Fig**). Likewise, we detected 9-fold less GA2 in Tay-Sachs sheep occipital lobe compared to Sandhoff cats. The difference in these levels may be attributed to the variation of ganglioside accumulation across species or differences in residual enzyme activity seen in these animal models (Sandhoff cats have < 1% normal levels and Tay Sachs sheep have relatively higher < 8% normal levels) [3, 30, 42]. Regardless, our results for both disease models demonstrate a progressive trend for the accumulation of these gangliosides in the brain.

### CNS accumulation of BMP phospholipids in an ovine model of Tay-Sachs disease

**Fig 7:**
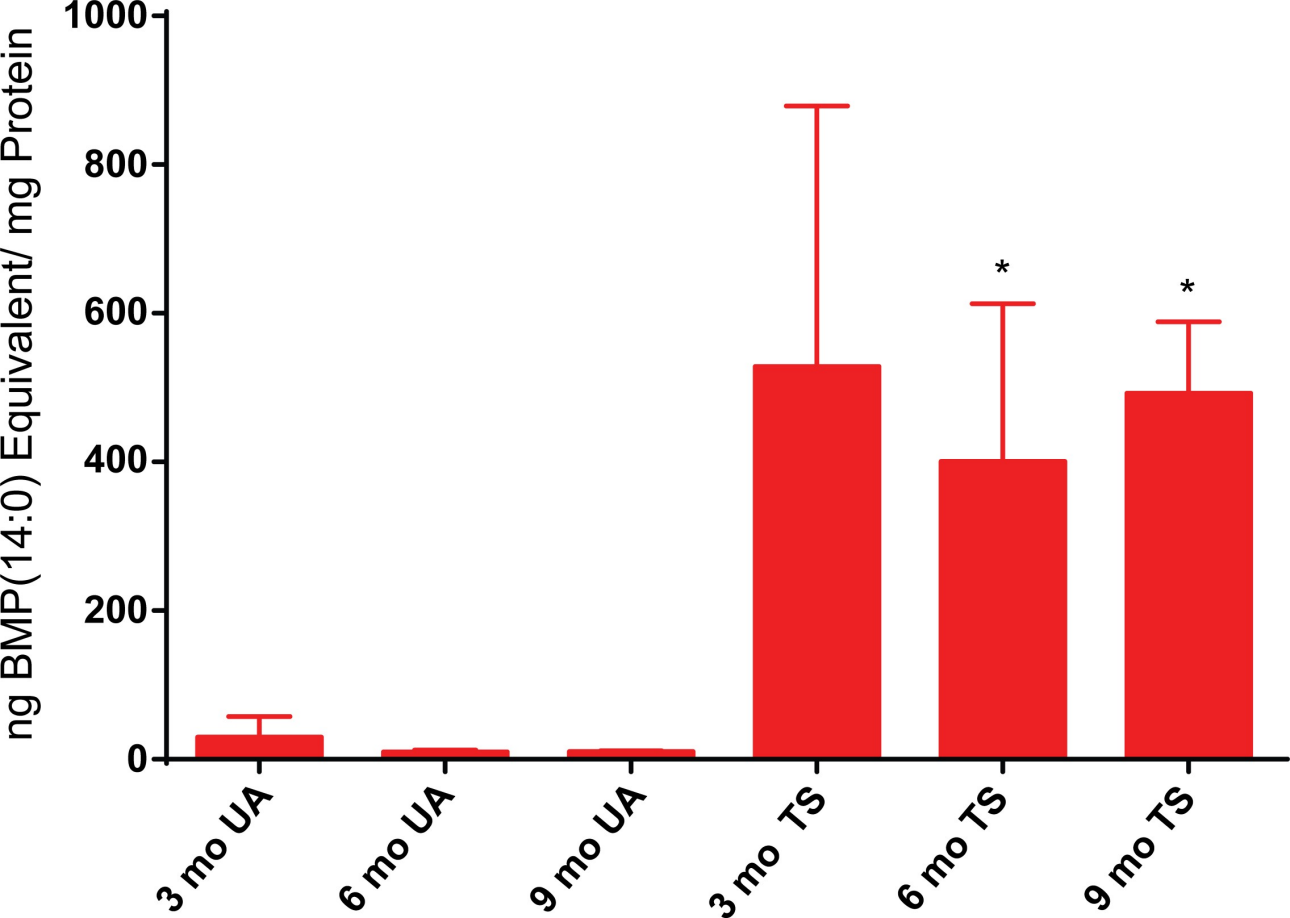
Ovine Tay-Sachs brain BMP phospholipid levels. Tissue samples from seven different brain regions from Tay-Sachs (TS) and age-matched unaffected (UA) normal control sheep were collected at three different ages: 3, 6 (n = 3), and 9 months old (n = 2) and BMP(22:6) levels measured by LC/MS/MS. The seven regions analyzed included two different samples from cerebellum and single biopsies from corona radiata, thalamus, occipital lobe, temporal lobe, and parietal lobe. The mean (± SD) for each set of three animals for each age tested corresponding to the cumulative results from the seven different brain regions, normalized to amount protein is shown. The mean results for each time point are shown as ng BMP(14:0) phospholipid equivalents/mg protein equivalents. Error bars represent ± SD. The degree of significant difference between age-matched UA and TS sheep is indicated by the number of asterisks above each TS bar (*** p ≤ 0.001, ** p ≤ 0.01, * p ≤ 0.05).

Similar to what we observed in the feline Sandhoff model, we detected an elevation in BMP phospholipid levels in the diseased sheep brain compared to the normal controls (**Fig 7 and S4 Table**). The unaffected animals showed low levels of BMP(22:6), but the three month old Tay-Sachs sheep exhibited large increases (29.9 ng BMP(22:6)/mg protein and 528.3 ng BMP(22:6) in unaffected and affected sheep, respectively). There was no significant further increase of BMP phospholipid observed at later time points suggesting that levels had peaked by 3 months of age (**S5 Table**). Similar to what we observed for ganglioside levels, the BMP phospholipid profiles showed differences among the 7 regions tested in Tay-Sachs sheep (**S3 Fig**). However, unlike the big differences in the levels of accumulation we saw in the occipital lobe of Sandhoff cats and Tay-Sachs sheep for GM2 and GA2 levels, the occipital lobe BMP(22:6) levels were comparable between these two models (**S2B Fig**). Interestingly, unlike in Sandhoff cats, we saw no increase in accumulation over time. This may be due to species differences or the time points chosen. We saw a significant increase in feline BMP(22:6) levels between the two and four month time points. Our earliest time point for ovines was three months. It is possible that BMP(22:6) may have peaked by then in the felines as well.

### Effects on oligosaccharide metabolites in an ovine model of Tay-Sachs disease

In Tay-Sachs sheep, the *HEXA* mutation only affects the production of the Hex A isozyme, leaving the Hex B homodimer largely unaffected [3, 42, 44]. With fully functional Hex B, the Tay-Sachs model should be able to effectively degrade oligosaccharide metabolites that enter the lysosome. Therefore, no significant accumulation of the kind of oligosaccharides present in Sandhoff disease are expected in the ovine Tay-Sachs model.

**Fig 8:**
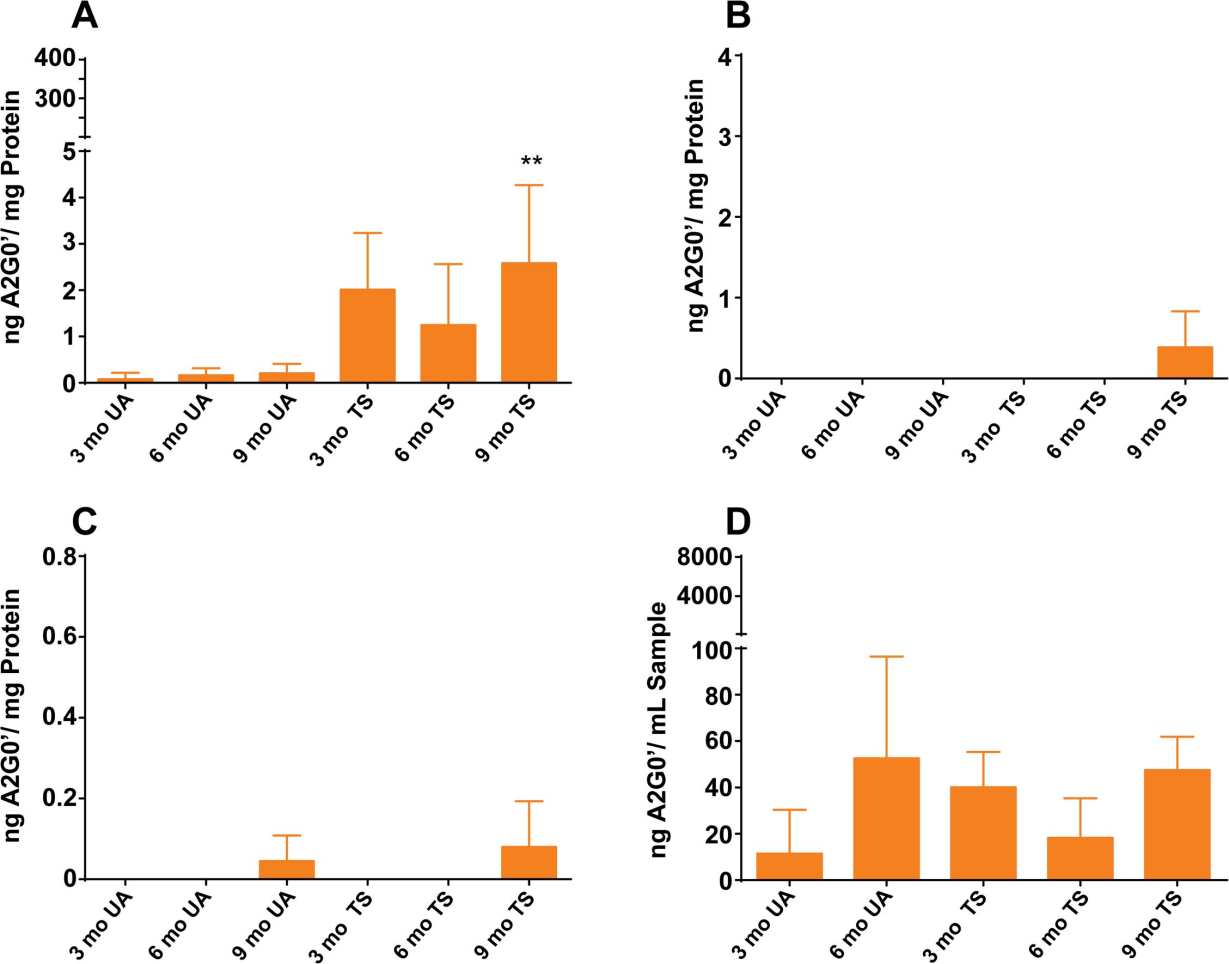
Ovine Tay-Sachs A2G0′ glycan metabolite levels. A2G0′ levels in Tay-Sachs (TS) and age-matched unaffected (UA) normal control sheep were measured in different sample matrices at 3, 6 (n = 3, unless otherwise indicated), and 9 months of age (n = 2, unless otherwise indicated). Where levels were detected, the mean results ± SD for each time point are shown as ng A2G0′/mg protein equivalents or ng A2G0′/mL sample. Error bars represent ± SD. The degree of significant difference between age-matched UA and TS sheep is indicated by the number of asterisks above each TS bar (*** p ≤ 0.001, ** p ≤ 0.01, * p ≤ 0.05). For comparison, results were scaled to the same scales (y-axes) used for the Sandhoff cats. (A) The cumulative A2G0′ levels from seven different brain regions including cerebellum, corona radiata, thalamus, occipital lobe, temporal lobe, and parietal lobe, normalized to amount protein. (B) CSF A2G0′ levels. (C) Serum A2G0′ levels. Six-month TS serum samples were collected n = 2. (D) Urine A2G0′ levels scaled to Sandhoff levels and without creatinine normalization. The six-month TS urine was collected n = 2 and no nine-month UA urine was collected.

We checked Tay-Sachs sheep for the presence of accumulating glycan metabolites and found only very low levels compared to those we detected in Sandhoff cats (**S4 Fig**). We detected no significant accumulation of the A2G0′ N-glycan metabolite in any of the brain samples we analyzed except for the last time point which showed about a 10-fold increase compared to age-matched unaffected controls (**Fig 8A and S4 Table**). In comparing the amounts accumulating in the occipital lobe of Sandhoff and Tay-Sachs animals, A2G0′ levels were far higher in Sandhoff cats (261.2 ng compared to 3.1 A2G0′/mg protein). As we did for gangliosides, we tested the same seven regions of the brain, including the occipital lobe. As with the occipital lobe, we did see signs of modest accumulation in some of these other regions including the corona radiate, parietal, temporal, and the thalamus (**S5 Fig**). Similar to the occipital lobe, these increases were significant only at the nine-month time point.

As we did with Sandhoff cats, we also analyzed A2G0′ levels in CSF, urine and, for sheep, instead of plasma we analyzed serum (**Fig 8**). No significant amounts were detected in CSF and serum collected from Tay-Sachs sheep at any of the time points tested (**Fig 8B and 8C**). Similar to the normal control cats that we compared to Sandhoff cats, urine from age-matched unaffected sheep exhibited only small measurable amounts of A2G0′ which were not perturbed by disease. There was no significant increase due to Hex A deficiency and no age-related increases were detected. What’s more, the urinary levels detected in both unaffected and affected sheep were similar to the normal range seen in normal cats (compare **S2** and **S4 Tables**).

The results with A2G0′ are fairly consistent with a deficiency in Hex A but not Hex B having little to no effect on the degradation of glycans. While on the other hand, Hex B deficiency leads to accumulation of both glycosphingolipids and glycans. The small increase in A2G0′ levels we detected in some of the brain regions we tested could be due to lysosomal dysfunction brought on by chronic disease which might affect a number of catabolic processes including glycan degradation. However, the low levels detected in Tay-Sachs sheep compared to Sandhoff cats suggests that its accumulation is not clinically meaningful in Tay-Sachs disease.

## Discussion

GM2 gangliosidoses cause neurodegeneration and early mortality in patients that suffer from infantile or juvenile onset forms. Therapeutic research is ongoing as there are no currently approved treatments for these diseases. Mouse models have helped enormously in our understanding of GM2 gangliosidoses [3, 25, 35, 45], but their translatability to human patients has not been established and may be somewhat limited. Naturally occurring large animal models that better approximate human pathology can provide a better understanding of disease progression in humans and be a better vehicle for preclinical testing of newly developed therapeutics.

This paper presents the first longitudinal natural history study of primary and secondary storage products in large animal models of Sandhoff and Tay-Sachs diseases. Also, this is the first report of the longitudinal accumulation of a glycan metabolite (A2G0′) in a large animal model of Sandhoff disease. Both lysosomal storage disorders result in a large accumulation of GM2 ganglioside with marginal accumulation of GA2 ganglioside in the brain of affected animals. Consequently, they have come to be known as GM2 gangliosidoses. However, a number of studies have shown that other compounds accumulate in these disorders. For instance, both Sandhoff and Tay-Sachs show disease-related increases in a biomarker for phospholipidosis, BMP(22:6) phospholipid, known to be elevated in both GM1 and GM2 gangliosidoses [19]. In Sandhoff disease, storage of soluble oligosaccharide metabolites results from incomplete degradation of glycan moieties from glycoproteins [14, 36–38]. While BMP(22:6) is a secondary biomarker present in elevated levels presumably due to lysosomal dysfunction, both gangliosides and free glycan limit digestion products are primary biomarkers of this disease, since both possess a β-linked N-acetylhexosamine at their non-reducing ends which is the catalytic target of the Hex B enzyme deficient in Sandhoff disease. On the other hand, in Tay-Sachs disease only Hex A is affected and not Hex B. Therefore, glycans entering the lysosome should be degraded normally. Thus, only gangliosides and BMP phospholipid accumulate in this type of GM2 gangliosidosis.

For this study, we analyzed gangliosides, a representative oligosaccharide metabolite A2G0′, and BMP phospholipid in a feline model of Sandhoff disease and a ovine model of Tay-Sachs disease in order to evaluate the effects of disease and age on the accumulation of these biochemical species. We looked at four matrices, brain, CSF, plasma (in Sandhoff) or serum (in Tay-Sachs), and urine, at three different time points over the life span of these animals and compared biomarker levels to those of age-matched unaffected normal controls.

For the Sandhoff cats, we observed highly elevated levels of GM2 gangliosides and the corresponding asialo GA2 gangliosides in the brain (occipital lobe) at all three time points. The accumulation of GM2 and GA2 did not show signs of reaching a plateau at the last point tested (four months) suggesting that these disease-related gangliosides could continue to rise if the animals had lived longer. Very low levels of GM2 and GA2 were detected in wild type age-matched brain samples at all of the three time points. Low levels of BMP(22:6) were detected in normal control cats at later time points, while in affected cats BMP(22:6) levels were greatly elevated compared to controls with increasing accumulation up to 4 months of age.

We detected a number of soluble oligosaccharides in Sandhoff cat brain (occipital lobe) at all ages tested. These glycan species correspond to limit digestion products that accumulate due to Hex β-subunit deficiency. Product ion analysis of these species showed them to be structurally consistent with substrates of Hex B since they contained HexNAc residues at their non-reducing ends. One of these, A2G0′, was chosen as a quantifiable biomarker for this class of metabolite since a standard for GRIL-LC/MS analysis could be easily generated. A2G0′ was not detected in the occipital lobe, CSF, or plasma of control animals at any age. However, high levels of this metabolite were detected in Sandhoff cats at all time points tested and A2G0′ levels showed significant increase with age in the brain which, like the GM2 and GA2 gangliosides, had not peaked by the last time point. In CSF and plasma, however, A2G0′ levels had peaked by the first time point. The urinary levels of A2G0′ were highly elevated in affected animals, reaching almost 5 μg per ml of urine while remaining at very low levels in unaffected animals. There was a good correlation between GM2/GA2 and A2G0′ levels in the occipital lobe of affected animals demonstrating the disease-relatedness of this metabolite similar to that of GM2 and GA2.

For Tay-Sachs sheep, we combined measurements from 7 different brain regions to get the total brain result. We observed increases in both GM2 and GA2 gangliosides, with GM2 being the most abundant ganglioside across the three time points tested compared to age-matched unaffected controls. We observed an age-dependent increase in accumulation by the final time point, but this was significant for GA2 only. BMP(22:6) was also increased in the Tay-Sachs sheep compared to age-matched wild type sheep, but the levels plateaued by the first time point in affected animals.

Surprisingly, we observed a modest but significant amount of A2G0′ in Tay-Sachs sheep brain that was detectable only at the terminal nine-month time point. This was unexpected since the Hex B enzyme is not compromised in Tay-Sachs disease and lysosomal degradation of N-glycans should proceed normally. While this accumulation was significant, it was at levels 85-fold less than those detected in Sandhoff cats comparing the occipital lobes from both models. A possible explanation may be that this low level of accumulation is due to secondary storage of glycan metabolites resulting from lysosomal dysfunction caused by chronic disease in the Tay-Sachs sheep.

As expected, GM2 and GA2 ganglioside levels were elevated in both Sandhoff cats and Tay-Sachs sheep. BMP(22:6) phospholipid was also elevated in both disease animals. Comparing the occipital lobe from affected cats and sheep showed GM2 levels to be 18-fold higher and GA2 levels 9-fold higher in Sandhoff cats compared to Tay Sachs sheep while BMP(22:6) levels were comparable. Both ganglioside and BMP phospholipid profiles showed differences among the 7 regions tested in the Tay-Sachs sheep. For example, at the 9-month time point, GM2 levels were as high as 52.1 ng/μg protein in the temporal lobe and as low as 2.0 ng/μg protein in the occipital lobe, a difference of 26-fold across brain regions. These differences as well as those between Sandhoff cats and Tay-Sachs sheep may be due to variations in disease impact and differences in residual enzyme activity across tissues and species. Previous studies have shown varying pathology in different brain regions of the Tay-Sachs sheep [43], which may be explained by the different accumulation patterns in the brain regions observed in this study. Overall, the increase in ganglioside accumulation in the brains of these two large animal models is consistent with progressing pathology of these diseases.

The lack of appreciable oligosaccharide metabolite accumulation in Tay-Sachs, especially early on in the progression of the disease, is one of the significant differences between Tay-Sachs and Sandhoff disease. This difference may underpin to some degree the clinical differences between these two disorders such as the hepatosplenomegaly and bone abnormalities observed in Sandhoff patients but not in Tay-Sachs.

While we found disease-appropriate changes in these biomarkers, some of our results showed relatively high standard deviations. This was expected because of the variability of the response to disease within any population. We made no attempt to reduce the variability by normalizing analyte measurements to residual β-hexosaminidase enzyme activity but instead normalized to protein amount or sample volume. For future studies, a comparison could be made between residual enzyme activity, disease severity and/or biomarker levels.

A related lysosomal metabolic disorder α-mannosidosis exhibits similar symptoms to those of Sandhoff and Tay-Sachs disease including severe neuropathology. However, unlike these two GM2 gangliosidoses, α-mannosidosis is characterized by the accumulation of oligosaccharides with glycosphingolipid levels remaining in the normal range [46, 47]. This makes the accumulation of oligosaccharide metabolites in Sandhoff disease an interesting clinical question regarding their possible role in pathogenesis. Determining the importance of oligosaccharide accumulation in Sandhoff disease progression should remain an active area of inquiry until this is better understood. Any treatment developed for this particular disorder should be evaluated by its effects on both ganglioside and oligosaccharide metabolite accumulation.

Along these lines, our method for measuring levels of A2G0′ may have application in testing biochemical efficacy of new therapeutics. While GM2 and GA2 gangliosides accumulate mostly in the brain and are challenging to measure in other sample matrices, we saw good correlation between GM2 and GA2 ganglioside accumulation with A2G0′ glycan metabolite accumulation in the occipital lobe of Sandhoff cats. We detected significant disease-related elevation of A2G0′ levels in multiple liquid matrices including urine from the same animals suggesting that this glycan metabolite may be a useful clinical tool since it produces a measurable signal in non-invasive sample types. To fully validate a urinary A2G0′ assay, a study of human patients would have to be undertaken in order to explore its limitations and reliability. Currently, enzyme-replacement therapies [5], gene therapies and substrate reduction therapies [48] are being studied as possible intervention strategies for GM2 gangliosidoses [49–52]. Urinary A2G0′ analysis may help support such efforts.

This natural history study involving two large animal models which mimic GM2 gangliosidoses in humans has led to a better understanding of how disease progression influences the accumulation of pathology-related metabolites. These metabolites are useful for evaluating disease severity or as pharmacodynamic markers for measuring drug efficacy. The soluble A2G0′ metabolite, in the future, may be important in these roles, especially since its levels can be monitored in an easily accessible sample matrix such as urine. However, there is still much we don’t understand about what drives the different aspects of these disease. The importance of β-hexosaminidase substrates in driving disease progression should be explored more fully. These two large animal models may provide a valuable resource to that end.

## Supporting information

S1 Fig Tay-Sachs ganglioside levels in different brain regions. Brain samples from Tay-Sachs (TS) and age-matched unaffected (WT) normal control sheep were provided as frozen tissue from different brain regions and were analyzed for ganglioside levels. The brain samples tested included: (**A-B**) cerebellum, (**C**) occipital cortex, (**D**) temporal lobe, (**E**) parietal cortex, (**F**) corona radiata, and (**G**) thalamus. Samples for each time point were collected in triplicate for analysis except as noted in **S1 Table**. Bars represent GM1 (purple), GA1 (pink), GM2 (blue), GA2 (green), and GM3 (orange) ganglioside levels (means ± SD).
(DOCX)

S2 Fig Comparison of occipital brain region in Sandhoff and Tay-Sachs for glycolipid, phospholipid, and glycan biomarkers. Results for Sandhoff (SH, blue bars) and Tay-Sachs (TS, red bars) occipital lobe biopsies were compared. Three animals for each time point were analyzed except as indicated in **S1 Table**. (**A**) Sandhoff vs. Tay-Sachs GM2 levels. (**B**) Sandhoff vs. Tay-Sachs BMP(22:6) levels. (**C**) Sandhoff vs. Tay-Sachs A2G0′ levels. Bars represents means ± SD.
(DOCX)

S3 Fig Tay-Sachs BMP levels in 7 different brain regions. BMP(22:6) phospholipid levels were measured in Tay-Sachs (TS) and age-matched unaffected wild type (WT) normal controls. Brain samples from Tay-Sachs sheep were collected in triplicate for each time point except as indicated in **S1 Table**. (**A-B**) cerebellum, (**C**) occipital cortex, (**D**) temporal lobe, (**E**) parietal cortex, (**F**) corona radiata, and (**G**) thalamus. Bars represent the mean ± SD of BMP(22:6) levels.
(DOCX)

S4 Fig Lack of substantial glycan metabolites in Tay-Sachs sheep brain. The extracted ion current for glycan metabolites in Sandhoff cat (top) and Tay-Sachs sheep (bottom) is shown along with putative structures and the A2G0′ species which was verified by data-dependent product ion analysis and co-elution with differentially isotope labeled A2G0′ standard. Other than a few oligoglucosides (*m/z* = 744.28 and 906.33) only very low levels of the types of oligosaccharides that are so prominent in Sandhoff cats are found in the Tay-Sachs ovine sample. Monosaccharides within the oligosaccharides are shown symbolically as follows: glucose (blue circles), galactose (yellow circles), mannose (green circles), generic hexose (open circles), N-acetylglucosamine (blue squares), N-acetylgalactosamine (yellow squares), and generic N-acetylhexosamine (open squares). The *m/z* values for each species along with corresponding putative structures are shown where they eluted. The ion intensity (relative abundance) for the Tay-Sachs sample was scaled to that of the Sandhoff sample for easier comparison.
(DOCX)

S5 Fig A2G0′ glycan levels in individual brain regions in Tay-Sachs sheep. A2G0′ levels were measured in Tay-Sachs (TS) and age-matched unaffected wild type (WT) normal controls. Brain samples from Tay-Sachs sheep were collected in triplicate for each time point except as indicated in S1 Table. (**A-B**) cerebellum, (**C**) occipital cortex, (**D**) temporal lobe, (**E**) parietal cortex, (**F**) corona radiata, and (**G**) thalamus. Bars represent means ± SD of A2G0′ levels in the regions tested.
(DOCX)

S1 Table Samples analyzed. Brain samples from Sandhoff feline were provided as powdered brain from a single punch (occipital lobe). Brain samples from Tay-Sachs (TS) sheep were provided as frozen tissue from seven different brain regions including: cerebellum sample 1 and 2, occipital lobe, temporal lobe, parietal lobe, corona radiata, and thalamus. Additional samples taken from each animal included cerebrospinal fluid (CSF), plasma or serum, and urine. Samples for each time point, sample matrix, and genotype were collected in triplicate except where noted in this table (colored cells).
(DOCX)

S2 Table Sandhoff feline analyte measurement results. Quantitative results for each analyte tested are shown for Sandhoff (SH) and age-matched unaffected (UA) cats at 1, 2, and 4 months of age. The number of animals (n) in each group is shown along with the mean and standard deviation (SD) and the p-value from one-tailed t-tests. Units are ng/μg protein for gangliosides, ng/mg protein for BMP phospholipid and A2G0′ glycan metabolite except for urinary A2G0′ levels which are expressed as ng/mL of urine.
(DOCX)

S3 Table Age effect on analyte accumulation in Sandhoff felines. A two-way ANOVA with Tukey HSD post hoc test was carried out on analyte levels in Sandhoff felines to determine statistical differences between age groups suggesting different levels of accumulation as a function of age. The analytes tested, the age comparisons, and the adjusted p values (p*) are shown. Resulting p-values less than 0.05 are significant.
(DOCX)

S4 Table Tay-Sachs ovine analyte measurement results. Quantitative results for each analyte tested are shown for Tay-Sachs (TS) and age-matched unaffected (UA) sheep at 3, 6, and 9 months of age. The number of animals (n) in each group is shown along with the mean and standard deviation (SD) and the p-value from one-tailed t-tests. Units are ng/μg protein for gangliosides, ng/mg protein for BMP phospholipid and A2G0′ glycan metabolite except for urinary A2G0′ levels which are expressed as ng/mL of urine.
(DOCX)

S5 Table Age effect on analyte accumulation in Tay-Sachs sheep. A two-way ANOVA with Tukey HSD post hoc test was carried out on analyte levels in Tay-Sachs ovines to determine statistical differences between age groups suggesting different levels of accumulation as a function of age. The analytes tested, the age comparisons, and the adjusted p values (p*) are shown. Resulting p-values less than 0.05 are significant.
(DOCX)

## Abbreviations

A2G0: Oxford glycan naming designation for core (G0) heptasaccharide
A2G0′: hexasaccharide product glycan generated by enzymatic removal of reducing end GlcNAc from A2G0
BMP: bis-(monoacylglycero)-phosphate
BMP(22:6)–: di-22:6-bis(monoacylglycerol)phosphate
GlcNAc: N-acetylglucosamine
GRIL-LC/MS: glycan reductive isotope labeling liquid chromatography mass spectrometry
GSLs: glycosphingolipids
Hex: β-N*-*acetylhexosaminidase
HexNAc: *N-*acetylhexosamine
LC/MS: liquid chromatography/mass spectrometry
LC-MS/MS: liquid chromatography/tandem mass spectrometry
NaBH_3_CN: sodium cyanoborohydride
