# Supplementary material for: Natural history study of glycan accumulation in large animal models of GM2 gangliosidoses
Source: PLoS One. 2020 Dec 1;15(12):e0243006. doi: 10.1371/journal.pone.0243006 (PMC7707493; doi:10.1371/journal.pone.0243006)
Supplement: S1 Fig — Brain samples from Tay-Sachs (TS) and age-matched unaffected (WT) normal control sheep were provided as frozen tissue from different brain regions and were analyzed for ganglioside levels. The brain samples tested included: (A-B) cerebellum, (C) occipital cortex, (D) temporal lobe, (E) parietal cortex, (F) corona radiata, and (G) thalamus. Samples for each time point were collected in triplicate for analysis except as noted in S1 Table. Bars represent GM1 (purple), GA1 (pink), GM2 (blue), GA2 (green), and GM3 (orange) ganglioside levels (means ± SD). (DOCX) [file pone.0243006.s001.docx]

**S1 Fig. Tay-Sachs ganglioside levels in different brain regions.** Brain samples from Tay-Sachs (TS) and age-matched unaffected (WT) normal control sheep were provided as frozen tissue from different brain regions and were analyzed for ganglioside levels. The brain samples tested included: (**A-B**) cerebellum, (**C**) occipital cortex, (**D**) temporal lobe, (**E**) parietal cortex, (**F**) corona radiata, and (**G**) thalamus. Samples for each time point were collected in triplicate for analysis except as noted in **S1 Table**. Bars represent GM1 (purple), GA1 (pink), GM2 (blue), GA2 (green), and GM3 (orange) ganglioside levels (means ± SD).
