# Supplementary material for: Natural history study of glycan accumulation in large animal models of GM2 gangliosidoses
Source: PLoS One. 2020 Dec 1;15(12):e0243006. doi: 10.1371/journal.pone.0243006 (PMC7707493; doi:10.1371/journal.pone.0243006)
Supplement: S2 Fig — Results for Sandhoff (SH, blue bars) and Tay-Sachs (TS, red bars) occipital lobe biopsies were compared. Three animals for each time point were analyzed except as indicated in S1 Table. (A) Sandhoff vs. Tay-Sachs GM2 levels. (B) Sandhoff vs. Tay-Sachs BMP(22:6) levels. (C) Sandhoff vs. Tay-Sachs A2G0′ levels. Bars represents means ± SD. (DOCX) [file pone.0243006.s002.docx]

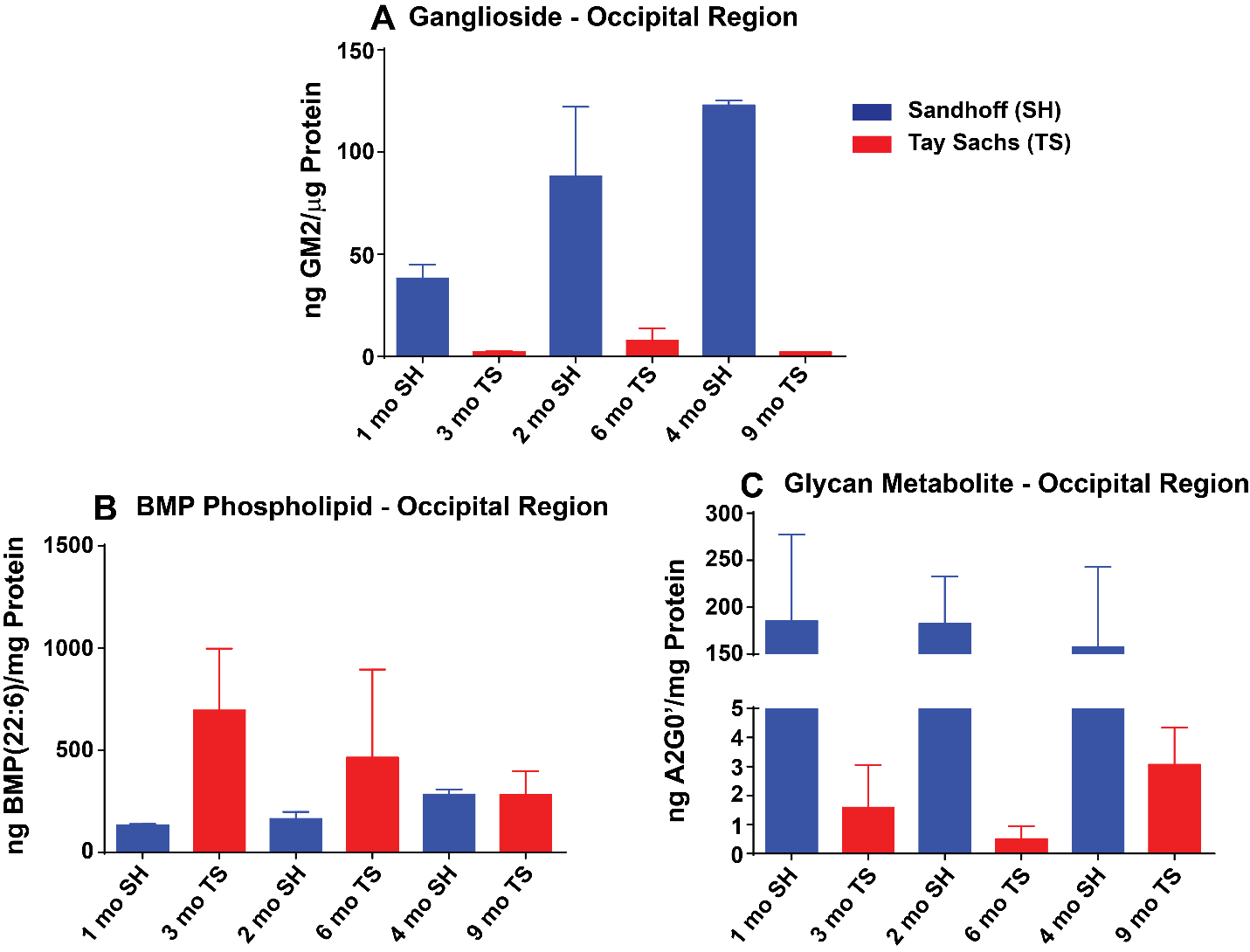


**S2 Fig. Comparison of occipital brain region in Sandhoff and Tay-Sachs for glycolipid, phospholipid, and glycan biomarkers.**  Results for Sandhoff (SH, blue bars) and Tay-Sachs (TS, red bars) occipital lobe biopsies were compared. Three animals for each time point were analyzed except as indicated in **S1 Table**. (**A**) Sandhoff vs. Tay-Sachs GM2 levels. (**B**) Sandhoff vs. Tay-Sachs BMP(22:6) levels. (**C**) Sandhoff vs. Tay-Sachs A2G0′ levels. Bars represents means ± SD.
