# Supplementary material for: Natural history study of glycan accumulation in large animal models of GM2 gangliosidoses
Source: PLoS One. 2020 Dec 1;15(12):e0243006. doi: 10.1371/journal.pone.0243006 (PMC7707493; doi:10.1371/journal.pone.0243006)
Supplement: S3 Fig — BMP(22:6) phospholipid levels were measured in Tay-Sachs (TS) and age-matched unaffected wild type (WT) normal controls. Brain samples from Tay-Sachs sheep were collected in triplicate for each time point except as indicated in S1 Table. (A-B) cerebellum, (C) occipital cortex, (D) temporal lobe, (E) parietal cortex, (F) corona radiata, and (G) thalamus. Bars represent the mean ± SD of BMP(22:6) levels. (DOCX) [file pone.0243006.s003.docx]

**

**

**S3 Fig. Tay-Sachs BMP levels in 7 different brain regions.** BMP(22:6) phospholipid levels were measured in Tay-Sachs (TS) and age-matched unaffected wild type (WT) normal controls. Brain samples from Tay-Sachs sheep were collected in triplicate for each time point except as indicated in **S1 Table**. (**A-B**) cerebellum, (**C**) occipital cortex, (**D**) temporal lobe, (**E**) parietal cortex, (**F**) corona radiata, and (**G**) thalamus. Bars represent the mean ± SD of BMP(22:6) levels.
