# Supplementary material for: Natural history study of glycan accumulation in large animal models of GM2 gangliosidoses
Source: PLoS One. 2020 Dec 1;15(12):e0243006. doi: 10.1371/journal.pone.0243006 (PMC7707493; doi:10.1371/journal.pone.0243006)
Supplement: S4 Fig — The extracted ion current for glycan metabolites in Sandhoff cat (top) and Tay-Sachs sheep (bottom) is shown along with putative structures and the A2G0′ species which was verified by data-dependent product ion analysis and co-elution with differentially isotope labeled A2G0′ standard. Other than a few oligoglucosides (m/z = 744.28 and 906.33) only very low levels of the types of oligosaccharides that are so prominent in Sandhoff cats are found in the Tay-Sachs ovine sample. Monosaccharides within the oligosaccharides are shown symbolically as follows: glucose (blue circles), galactose (yellow circles), mannose (green circles), generic hexose (open circles), N-acetylglucosamine (blue squares), N-acetylgalactosamine (yellow squares), and generic N-acetylhexosamine (open squares). The m/z values for each species along with corresponding putative structures are shown where they eluted. The ion intensity (relative abundance) for the Tay-Sachs sample was scaled to that of the Sandhoff sample for easier comparison. (DOCX) [file pone.0243006.s004.docx]

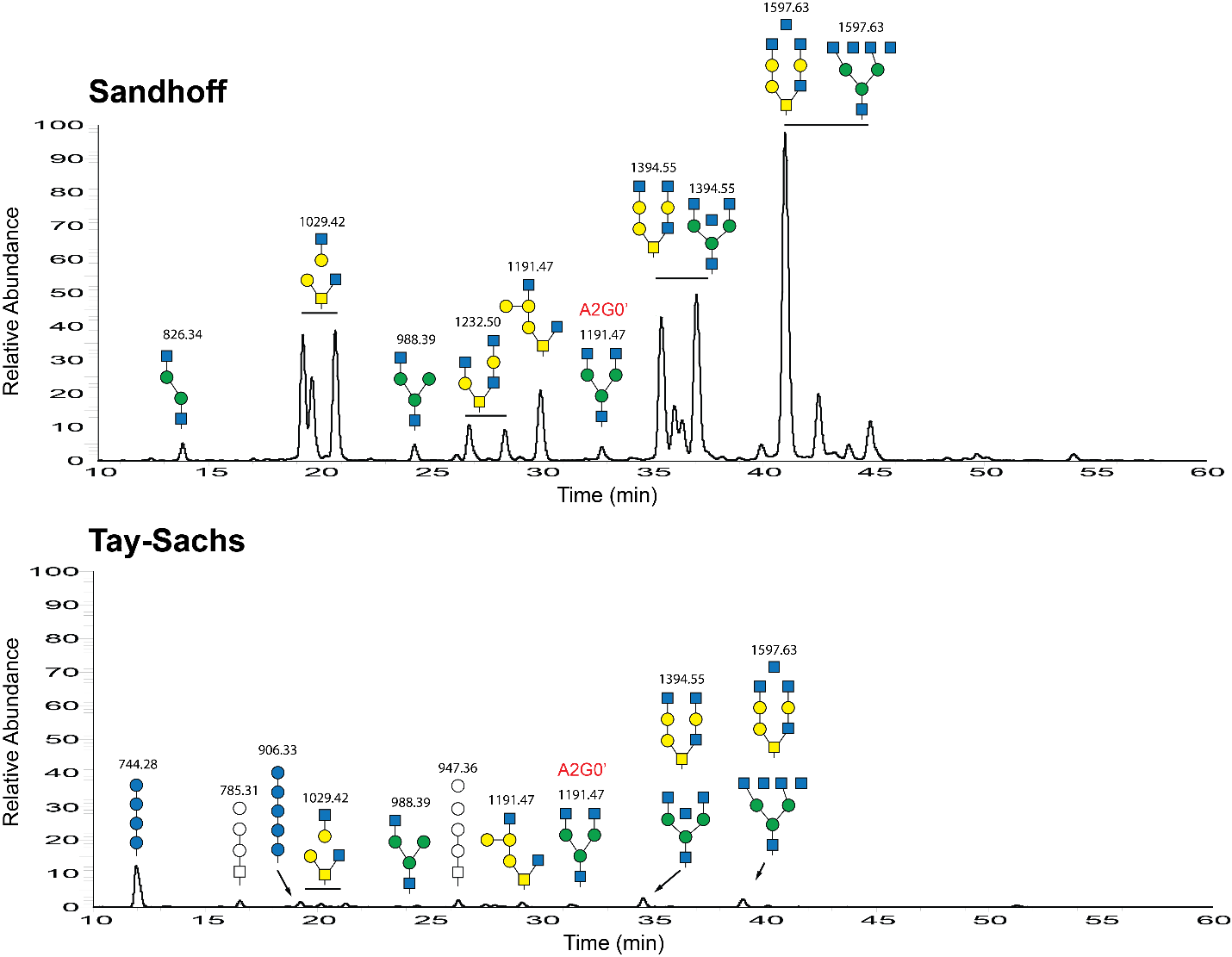


**S4 Fig. Lack of substantial glycan metabolites in Tay-Sachs sheep brain.** The extracted ion current for glycan metabolites in Sandhoff cat (top) and Tay-Sachs sheep (bottom) is shown along with putative structures and the A2G0′ species which was verified by data-dependent product ion analysis and co-elution with differentially isotope labeled A2G0′ standard. Other than a few oligoglucosides (*m/z* = 744.28 and 906.33) only very low levels of the types of oligosaccharides that are so prominent in Sandhoff cats are found in the Tay-Sachs ovine sample. Monosaccharides within the oligosaccharides are shown symbolically as follows: glucose (blue circles), galactose (yellow circles), mannose (green circles), generic hexose (open circles), N-acetylglucosamine (blue squares), N-acetylgalactosamine (yellow squares), and generic N-acetylhexosamine (open squares). The *m/z* values for each species along with corresponding putative structures are shown where they eluted. The ion intensity (relative abundance) for the Tay-Sachs sample was scaled to that of the Sandhoff sample for easier comparison.
