# Supplementary material for: Natural history study of glycan accumulation in large animal models of GM2 gangliosidoses
Source: PLoS One. 2020 Dec 1;15(12):e0243006. doi: 10.1371/journal.pone.0243006 (PMC7707493; doi:10.1371/journal.pone.0243006)
Supplement: S1 Table — Brain samples from Sandhoff feline were provided as powdered brain from a single punch (occipital lobe). Brain samples from Tay-Sachs (TS) sheep were provided as frozen tissue from seven different brain regions including: cerebellum sample 1 and 2, occipital lobe, temporal lobe, parietal lobe, corona radiata, and thalamus. Additional samples taken from each animal included cerebrospinal fluid (CSF), plasma or serum, and urine. Samples for each time point, sample matrix, and genotype were collected in triplicate except where noted in this table (colored cells). (DOCX) [file pone.0243006.s006.docx]

**S1 Table. Samples analyzed.** Brain samples from Sandhoff feline were provided as powdered brain from a single punch (occipital lobe). Brain samples from Tay-Sachs (TS) sheep were provided as frozen tissue from seven different brain regions including: cerebellum sample 1 and 2, occipital lobe, temporal lobe, parietal lobe, corona radiata, and thalamus. Additional samples taken from each animal included cerebrospinal fluid (CSF), plasma or serum, and urine. Samples for each time point, sample matrix, and genotype were collected in triplicate except where noted in this table (colored cells).
