# Supplementary material for: Natural history study of glycan accumulation in large animal models of GM2 gangliosidoses
Source: PLoS One. 2020 Dec 1;15(12):e0243006. doi: 10.1371/journal.pone.0243006 (PMC7707493; doi:10.1371/journal.pone.0243006)
Supplement: S2 Table — Quantitative results for each analyte tested are shown for Sandhoff (SH) and age-matched unaffected (UA) cats at 1, 2, and 4 months of age. The number of animals (n) in each group is shown along with the mean and standard deviation (SD) and the p-value from one-tailed t-tests. Units are ng/μg protein for gangliosides, ng/mg protein for BMP phospholipid and A2G0′ glycan metabolite except for urinary A2G0′ levels which are expressed as ng/mL of urine. (DOCX) [file pone.0243006.s007.docx]

| **Analyte** | **Age/Disease** | **n** | **Mean** | **SD** | **p-value** |
| --- | --- | --- | --- | --- | --- |
| GM1 | 1 mo UA | 3 | 8.82 | 2.238593 | 0.2878 |
|  | 1 mo SH | 3 | 10.04 | 2.653871 |  |
|  | 2 mo UA | 3 | 10.57 | 1.735809 | 0.856 |
|  | 2 mo SH | 3 | 9.13 | 0.7496 |  |
|  | 4 mo UA | 3 | 10.63 | 0.708872 | 0.9013 |
|  | 4 mo SH | 3 | 7.14 | 3.22619 |  |
| GA1 | 1 mo UA | 3 | 0 | 0 | 0.0099 |
|  | 1 mo SH | 3 | 0.07 | 0.017321 |  |
|  | 2 mo UA | 3 | 0.033 | 0.005774 | 0.0365 |
|  | 2 mo SH | 3 | 0.167 | 0.066583 |  |
|  | 4 mo UA | 3 | 0.05 | 0.01 | 0.1386 |
|  | 4 mo SH | 3 | 0.213 | 0.191398 |  |
| GM2 | 1 mo UA | 3 | 0.167 | 0.10504 | 0.005044 |
|  | 1 mo SH | 3 | 38.18 | 6.667976 |  |
|  | 2 mo UA | 3 | 0.173 | 0.119304 | 0.02321 |
|  | 2 mo SH | 3 | 88.183 | 34.04294 |  |
|  | 4 mo UA | 3 | 0.24 | 0.07 | 6.02E-05 |
|  | 4 mo SH | 3 | 122.927 | 2.3466 |  |
| GA2 | 1 mo UA | 3 | 0.0167 | 0.015275 | 0.006004 |
|  | 1 mo SH | 3 | 4.3067 | 0.822456 |  |
|  | 2 mo UA | 3 | 0.0233 | 0.005774 | 0.02475 |
|  | 2 mo SH | 3 | 6.5533 | 2.614504 |  |
|  | 4 mo UA | 3 | 0.02 | 0 | 0.01791 |
|  | 4 mo SH | 3 | 14.68 | 4.939605 |  |
| GM3 | 1 mo UA | 3 | 0.18 | 0.036056 | 0.2263 |
|  | 1 mo SH | 3 | 0.2 | 0.017321 |  |
|  | 2 mo UA | 3 | 0.28 | 0.087178 | 0.5392 |
|  | 2 mo SH | 3 | 0.2733 | 0.066583 |  |
|  | 4 mo UA | 3 | 0.2267 | 0.011547 | 0.05396 |
|  | 4 mo SH | 3 | 0.3433 | 0.073711 |  |
| BMP(22:6) | 1 mo UA | 3 | 0 | 0 | 0.000312 |
|  | 1 mo SH | 3 | 133.3 | 5.774 |  |
|  | 2 mo UA | 3 | 6.67 | 11.547 | 0.005161 |
|  | 2 mo SH | 3 | 163.3 | 35.119 |  |
|  | 4 mo UA | 3 | 13.33 | 11.547 | 0.00033 |
|  | 4 mo SH | 3 | 283.3 | 25.166 |  |
| A2G0' (brain) | 1 mo UA | 3 | 0 | 0 | 0.009971 |
|  | 1 mo SH | 3 | 134.2033 | 33.32501 |  |
|  | 2 mo UA | 3 | 0 | 0 | 0.004475 |
|  | 2 mo SH | 3 | 129.75 | 21.40357 |  |
|  | 4 mo UA | 3 | 0 | 0 | 0.001766 |
|  | 4 mo SH | 3 | 261.15 | 26.95531 |  |
| A2G0' (CSF) | 1 mo UA | 3 | 0 | 0 | 0.1117 |
|  | 1 mo SH | 3 | 1.0467 | 1.040064 |  |
|  | 2 mo UA | 3 | 0 | 0 | 0.08343 |
|  | 2 mo SH | 3 | 2.0367 | 1.655909 |  |
|  | 4 mo UA | 3 | 0 | 0 | 0.004358 |
|  | 4 mo SH | 3 | 3.1867 | 0.518684 |  |
| A2G0' (plasma) | 1 mo UA | 3 | 0 | 0 | 0.03183 |
|  | 1 mo SH | 3 | 0.42 | 0.192873 |  |
|  | 2 mo UA | 3 | 0 | 0 | 0.02337 |
|  | 2 mo SH | 3 | 0.4767 | 0.185023 |  |
|  | 4 mo UA | 3 | 0 | 0 | 0.01951 |
|  | 4 mo SH | 3 | 0.52 | 0.183303 |  |
| A2G0' (urine) | 1 mo UA | 3 | 4.6733 | 4.98479 | 0.07992 |
|  | 1 mo SH | 3 | 2726.24 | 2151.625 |  |
|  | 2 mo UA | 3 | 15.87 | 3.057761 | 0.03626 |
|  | 2 mo SH | 3 | 4871.9 | 2397.432 |  |
|  | 4 mo UA | 3 | 19.5033 | 5.329018 | 0.051 |
|  | 4 mo SH | 3 | 2567.39 | 1528.907 |  |

**S2 Table.** **Sandhoff feline analyte measurement results.** Quantitative results for each analyte tested are shown for Sandhoff (SH) and age-matched unaffected (UA) cats at 1, 2, and 4 months of age. The number of animals (n) in each group is shown along with the mean and standard deviation (SD) and the p-value from one-tailed t-tests. Units are ng/μg protein for gangliosides, ng/mg protein for BMP phospholipid and A2G0′ glycan metabolite except for urinary A2G0′ levels which are expressed as ng/mL of urine.
