# Supplementary material for: Natural history study of glycan accumulation in large animal models of GM2 gangliosidoses
Source: PLoS One. 2020 Dec 1;15(12):e0243006. doi: 10.1371/journal.pone.0243006 (PMC7707493; doi:10.1371/journal.pone.0243006)
Supplement: S3 Table — A two-way ANOVA with Tukey HSD post hoc test was carried out on analyte levels in Sandhoff felines to determine statistical differences between age groups suggesting different levels of accumulation as a function of age. The analytes tested, the age comparisons, and the adjusted p values (p*) are shown. Resulting p-values less than 0.05 are significant. (DOCX) [file pone.0243006.s008.docx]

| Analyte | Age comparison | p* |
| --- | --- | --- |
| GM1 | 1 mo:2 mo | 0.993635 |
|  | 1 mo:4 mo | 0.561444 |
|  | 2 mo:4 mo | 0.847461 |
| GA1 | 1 mo:2 mo | 0.713742 |
|  | 1 mo:4 mo | 0.343346 |
|  | 2 mo:4 mo | 0.980038 |
| GM2 | 1 mo:2 mo | 0.009996 |
|  | 1 mo:4 mo | 0.000106 |
|  | 2 mo:4 mo | 0.090887 |
| GA2 | 1 mo:2 mo | 0.832029 |
|  | 1 mo:4 mo | 0.001443 |
|  | 2 mo:4 mo | 0.009973 |
| GM3 | 1 mo:2 mo | 0.620506 |
|  | 1 mo:4 mo | 0.076466 |
|  | 2 mo:4 mo | 0.661898 |
| BMP(22:6) | 1 mo:2 mo | 0.428906 |
|  | 1 mo:4 mo | 0.000006 |
|  | 2 mo:4 mo | 0.000061 |
| A2G0' (brain) | 1 mo:2 mo | 0.999705 |
|  | 1 mo:4 mo | 0.000046 |
|  | 2 mo:4 mo | 0.000033 |
| A2G0' (CSF) | 1 mo:2 mo | 0.688843 |
|  | 1 mo:4 mo | 0.068105 |
|  | 2 mo:4 mo | 0.553124 |
| A2G0' (plasma) | 1 mo:2 mo | 0.994026 |
|  | 1 mo:4 mo | 0.932210 |
|  | 2 mo:4 mo | 0.998297 |
| A2G0' (urine) | 1 mo:2 mo | 0.497241 |
|  | 1 mo:4 mo | 0.999992 |
|  | 2 mo:4 mo | 0.426167 |

**S3 Table.** **Age effect on analyte accumulation in Sandhoff felines.** A two-way ANOVA with Tukey HSD post hoc test was carried out on analyte levels in Sandhoff felines to determine statistical differences between age groups suggesting different levels of accumulation as a function of age. The analytes tested, the age comparisons, and the adjusted p values (p*) are shown. Resulting p-values less than 0.05 are significant.
