# Supplementary material for: Natural history study of glycan accumulation in large animal models of GM2 gangliosidoses
Source: PLoS One. 2020 Dec 1;15(12):e0243006. doi: 10.1371/journal.pone.0243006 (PMC7707493; doi:10.1371/journal.pone.0243006)
Supplement: S4 Table — Quantitative results for each analyte tested are shown for Tay-Sachs (TS) and age-matched unaffected (UA) sheep at 3, 6, and 9 months of age. The number of animals (n) in each group is shown along with the mean and standard deviation (SD) and the p-value from one-tailed t-tests. Units are ng/μg protein for gangliosides, ng/mg protein for BMP phospholipid and A2G0′ glycan metabolite except for urinary A2G0′ levels which are expressed as ng/mL of urine. (DOCX) [file pone.0243006.s009.docx]

| **Analyte** | **Age/Disease** | **n** | **Mean** | **SD** | **p-value** |
| --- | --- | --- | --- | --- | --- |
| GM1 | 3 mo UA | 3 | 0.030333 | 0.024767 | 0.1046 |
|  | 3 mo TS | 3 | 0.095048 | 0.062736 |  |
|  | 6 mo UA | 3 | 0.012333 | 0.00486 | 0.000199 |
|  | 6 mo TS | 3 | 0.087048 | 0.007732 |  |
|  | 9 mo UA | 2 | 0.025857 | 0.028688 | 0.1758 |
|  | 9 mo TS | 2 | 0.056857 | 0.010102 |  |
| GA1 | 3 mo UA | 3 | 0.007381 | 0.003018 | 0.002538 |
|  | 3 mo TS | 3 | 0.036333 | 0.005886 |  |
|  | 6 mo UA | 3 | 0.010667 | 0.002929 | 0.009672 |
|  | 6 mo TS | 3 | 0.086762 | 0.0194 |  |
|  | 9 mo UA | 2 | 0.0115 | 0.003536 | 0.06107 |
|  | 9 mo TS | 2 | 0.1245 | 0.03182 |  |
| GM2 | 3 mo UA | 3 | 0.503571 | 0.86751 | 0.01221 |
|  | 3 mo TS | 3 | 5.443286 | 1.778637 |  |
|  | 6 mo UA | 3 | 0.006476 | 0.001528 | 0.01608 |
|  | 6 mo TS | 3 | 6.064667 | 1.92861 |  |
|  | 9 mo UA | 2 | 0.009093 | 0.002556 | 1.41E-01 |
|  | 9 mo TS | 2 | 15.63636 | 10.45659 |  |
| GA2 | 3 mo UA | 3 | 0.01381 | 0.021692 | 0.04084 |
|  | 3 mo TS | 3 | 0.465905 | 0.24055 |  |
|  | 6 mo UA | 3 | 0.00281 | 0.000577 | 0.00924 |
|  | 6 mo TS | 3 | 1.343619 | 0.320062 |  |
|  | 9 mo UA | 2 | 0.003929 | 0.000909 | 0.02619 |
|  | 9 mo TS | 2 | 1.634 | 0.190111 |  |
| GM3 | 3 mo UA | 3 | 0.006095 | 0.000812 | 0.02472 |
|  | 3 mo TS | 3 | 0.021095 | 0.006105 |  |
|  | 6 mo UA | 3 | 0.006381 | 0.000787 | 0.000275 |
|  | 6 mo TS | 3 | 0.015048 | 0.000929 |  |
|  | 9 mo UA | 2 | 0.008071 | 0.001515 | 0.1426 |
|  | 9 mo TS | 2 | 0.051286 | 0.029496 |  |
| BMP(22:6) | 3 mo UA | 3 | 29.90476 | 27.61186 | 0.06608 |
|  | 3 mo TS | 3 | 528.3333 | 350.7684 |  |
|  | 6 mo UA | 3 | 9.761905 | 2.516611 | 0.04279 |
|  | 6 mo TS | 3 | 400.9524 | 211.9138 |  |
|  | 9 mo UA | 2 | 10.35714 | 1.111168 | 0.04444 |
|  | 9 mo TS | 2 | 492.5714 | 96.16652 |  |
| A2G0' (brain) | 3 mo UA | 3 | 0.077619 | 0.070773 | 0.09836 |
|  | 3 mo TS | 3 | 2.009524 | 1.75536 |  |
|  | 6 mo UA | 3 | 0.160476 | 0.042626 | 0.1519 |
|  | 6 mo TS | 3 | 1.244286 | 1.369629 |  |
|  | 9 mo UA | 2 | 0.206429 | 0.093944 | 0.005998 |
|  | 9 mo TS | 2 | 2.683571 | 0.188899 |  |
| A2G0' (CSF) | 3 mo UA | 3 | 0 | 0 | NA |
|  | 3 mo TS | 3 | 0 | 0 |  |
|  | 6 mo UA | 3 | 0 | 0 | NA |
|  | 6 mo TS | 3 | 0 | 0 |  |
|  | 9 mo UA | 2 | 0 | 0 | 0.2183 |
|  | 9 mo TS | 2 | 0.385 | 0.445477 |  |
| A2G0' (serum) | 3 mo UA | 3 | 0 | 0 | NA |
|  | 3 mo TS | 3 | 0 | 0 |  |
|  | 6 mo UA | 3 | 0 | 0 | NA |
|  | 6 mo TS | 3 | 0 | 0 |  |
|  | 9 mo UA | 2 | 0.045 | 0.06364 | 0.374 |
|  | 9 mo TS | 2 | 0.08 | 0.113137 |  |
| A2G0' (urine) | 3 mo UA | 3 | 11.43667 | 18.89833 | 0.05706 |
|  | 3 mo TS | 3 | 40.02333 | 15.23129 |  |
|  | 6 mo UA | 3 | 52.57333 | 43.83632 | 0.8421 |
|  | 6 mo TS | 3 | 18.255 | 17.00592 |  |
|  | 9 mo UA | 0 |  |  | NA |
|  | 9 mo TS | 2 | 47.465 | 14.41791 |  |

**S4 Table.** **Tay-Sachs ovine analyte measurement results.** Quantitative results for each analyte tested are shown for Tay-Sachs (TS) and age-matched unaffected (UA) sheep at 3, 6, and 9 months of age. The number of animals (n) in each group is shown along with the mean and standard deviation (SD) and the p-value from one-tailed t-tests. Units are ng/μg protein for gangliosides, ng/mg protein for BMP phospholipid and A2G0′ glycan metabolite except for urinary A2G0′ levels which are expressed as ng/mL of urine.
