# Supplementary material for: Natural history study of glycan accumulation in large animal models of GM2 gangliosidoses
Source: PLoS One. 2020 Dec 1;15(12):e0243006. doi: 10.1371/journal.pone.0243006 (PMC7707493; doi:10.1371/journal.pone.0243006)
Supplement: S5 Table — A two-way ANOVA with Tukey HSD post hoc test was carried out on analyte levels in Tay-Sachs ovines to determine statistical differences between age groups suggesting different levels of accumulation as a function of age. The analytes tested, the age comparisons, and the adjusted p values (p*) are shown. Resulting p-values less than 0.05 are significant. (DOCX) [file pone.0243006.s010.docx]

| Analyte | Age comparison | p* |
| --- | --- | --- |
| GM1 | 3 mo:6 mo | 0.999607 |
|  | 3 mo:9 mo | 0.779735 |
|  | 6 mo:9 mo | 0.894891 |
| GA1 | 3 mo:6 mo | 0.010881 |
|  | 3 mo:9 mo | 0.000377 |
|  | 6 mo:9 mo | 0.101129 |
| GM2 | 3 mo:6 mo | 0.999913 |
|  | 3 mo:9 mo | 0.080047 |
|  | 6 mo:9 mo | 0.106889 |
| GA2 | 3 mo:6 mo | 0.002030 |
|  | 3 mo:9 mo | 0.000512 |
|  | 6 mo:9 mo | 0.570200 |
| GM3 | 3 mo:6 mo | 0.972083 |
|  | 3 mo:9 mo | 0.055369 |
|  | 6 mo:9 mo | 0.020343 |
| BMP(22:6) | 3 mo:6 mo | 0.953005 |
|  | 3 mo:9 mo | 0.999922 |
|  | 6 mo:9 mo | 0.992951 |
| A2G0' (brain) | 3 mo:6 mo | 0.927324 |
|  | 3 mo:9 mo | 0.971879 |
|  | 6 mo:9 mo | 0.628247 |
| A2G0' (CSF) | 3 mo:6 mo | 1.000000 |
|  | 3 mo:9 mo | 0.103197 |
|  | 6 mo:9 mo | 0.103197 |
| A2G0' (serum) | 3 mo:6 mo | 1.000000 |
|  | 3 mo:9 mo | 0.342901 |
|  | 6 mo:9 mo | 0.342901 |
| A2G0' (urine) | 3 mo:6 mo | 0.933866 |
|  | 3 mo:9 mo | 0.999445 |
|  | 6 mo:9 mo | 0.863765 |

**S5 Table.** **Age effect on analyte accumulation in Tay-Sachs sheep.** A two-way ANOVA with Tukey HSD post hoc test was carried out on analyte levels in Tay-Sachs ovines to determine statistical differences between age groups suggesting different levels of accumulation as a function of age. The analytes tested, the age comparisons, and the adjusted p values (p*) are shown. Resulting p-values less than 0.05 are significant.
